# Computational Modeling of the Mobility, Stability, and Al Positioning Ability of Cyclic Cationic Organic Structure-Directing Agents in AEI Zeolite

**DOI:** 10.1021/jacsau.5c00094

**Published:** 2025-03-07

**Authors:** Pau Ferri, Pieter Cnudde, Manuel Moliner, Veronique van Speybroeck, Mercedes Boronat

**Affiliations:** † Instituto de Tecnología Química, Universitat Politècnica de València-Consejo Superior de Investigaciones Científicas, Avenida de los Naranjos s/n, 46022 València, Spain; ‡ Center for Molecular Modeling, Ghent University, Technologiepark 46, 9052 Zwijnaarde, Belgium

**Keywords:** zeolite, molecular dynamics, DFT, OSDA, Al distribution

## Abstract

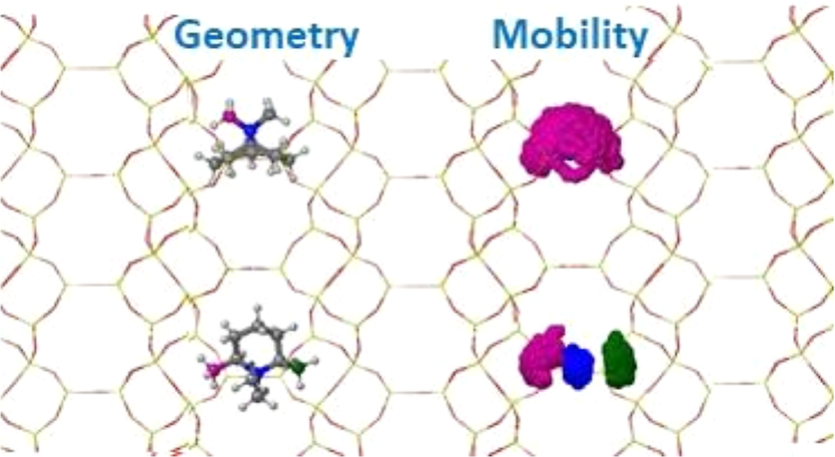

The stability and mobility of a set of organic structure-directing agents (OSDAs) with different molecular geometries and charge distribution confined within the pear-like cavities of neutral and Al-containing models of AEI zeolites have been investigated by using static density functional theory calculations and ab initio molecular dynamics simulations. The objective is to identify the role of electrostatic interactions between the OSDAs’ positive charge at N^+^ atoms and the anionic framework AlO_4_^–^ centers on the preferential stabilization of Al at specific crystallographic positions, opening the possibility to modulate the Al distribution in AEI zeolites. We find that several classical piperidinium-based OSDAs with diverse methyl-substituent patterns in the N-containing ring but a symmetrical charge distribution, as well as bulkier nonclassical azoniabicycle-heptane-based OSDAs with the positive charge asymmetrically located at one side of the molecule, behave similarly. All of them remain almost immobile at the center of the *aei* cavity along the simulations and always stabilize Al preferentially at the T1 crystallographic position. In contrast, an azabicyclo-octane-based OSDA with the positive charge located outside a cyclo-octane ring lacking substituents exhibits an enhanced mobility that includes full rotation within the *aei* cage and the ability to reach the regions of the cavity not accessible to the other OSDAs investigated. As a result, this highly mobile OSDA preferentially stabilizes Al in the T3 site, which might lead to differences in catalyst activity and stability for zeolite samples synthesized using this OSDA.

## Introduction

Zeolites are inorganic solids widely used in industrial and sustainable catalysis, adsorption, separation, and ion-exchange applications. They are composed by SiO_4_ and AlO_4_^–^ tetrahedral units linked to form microporous structures of channels and cavities of molecular dimensions that host the catalytic active sites where reactions take place. Shape-selectivity and confinement effects caused by the geometrical fitting of adsorbates into each zeolite topology, together with the possibility of modulating the amount and distribution of active sites in the framework, make these materials versatile and easily tunable for specific applications.^1−5^

The topological diversity of zeolites is derived from complex nucleation mechanisms and phase competition between metastable polymorphs during their crystallization. Zeolites are commonly synthesized in the presence of an organic structure-directing agent (OSDA) that strongly influences zeolite phase selectivity, imprinting a significant effect on the size and shape of the internal voids and channel systems.^6^ In addition, cationic OSDAs help modulating the distribution of the active sites favoring the siting of Al at specific crystallographic positions based on the proximity between the OSDA’s positive charge and the anionic lattice AlO_4_^–^ centers.^7−9^ Thus, designing the size, shape, and charge distribution of OSDAs is key for tailored zeolite synthesis,^10−13^ despite it being not clearly established whether Al positioning is kinetically or thermodynamically controlled during zeolite crystallization. It is important to note that the nature of Si and Al sources, inorganic cations, reaction conditions, and crystallization times is as crucial as the OSDAs used, making zeolite synthesis still reliant on trial-and-error methods. The well-defined crystalline structures of zeolites enable the connection of experimental data with theoretical modeling.^14−17^ Monte Carlo simulations of silica polymerization show that the OSDA volume and van der Waals interactions with lattice oxygen atoms are key to stabilizing microporous voids during crystallization.^18−20^ Initial studies quantified the affinity between OSDAs and targeted topologies by calculating the van der Waals interactions between silicate frameworks and organic guests. This led to fast OSDA screening methods^21−23^ successfully applied to design zeolites like AEI, ITE, STF, and STW.^24−27^ Further advancements include the design of OSDAs that mimic transition states to guide zeolite synthesis for specific reactions,^16,28^ and recent studies are applying high-throughput and machine learning techniques for fast screening of thousands of OSDAs.^29,30^

Predicting Al locations in specific tetrahedral sites in zeolites remains challenging due to the influence of various synthesis parameters, such as the inorganic cations in the gel, and to the difficulties in experimentally characterizing Al siting using techniques such as ^27^Al MAS NMR.^31−38^ Despite the great importance of controlling the Al distribution in zeolites for catalytic applications,^39,40^ few successful examples of synthesis–structure–activity relationships can be found in the literature,^41−44^ thus necessitating to improve the predictive character of computational studies. In this sense, it has been recently shown that combining static density functional theory (DFT) calculations of the thermodynamic stability of Al at different sites with ab initio molecular dynamics (AIMD) simulations of OSDA mobility provides a good description of the influence of OSDA on Al distribution in CHA and MFI frameworks.^45−48^

**Figure 1:**
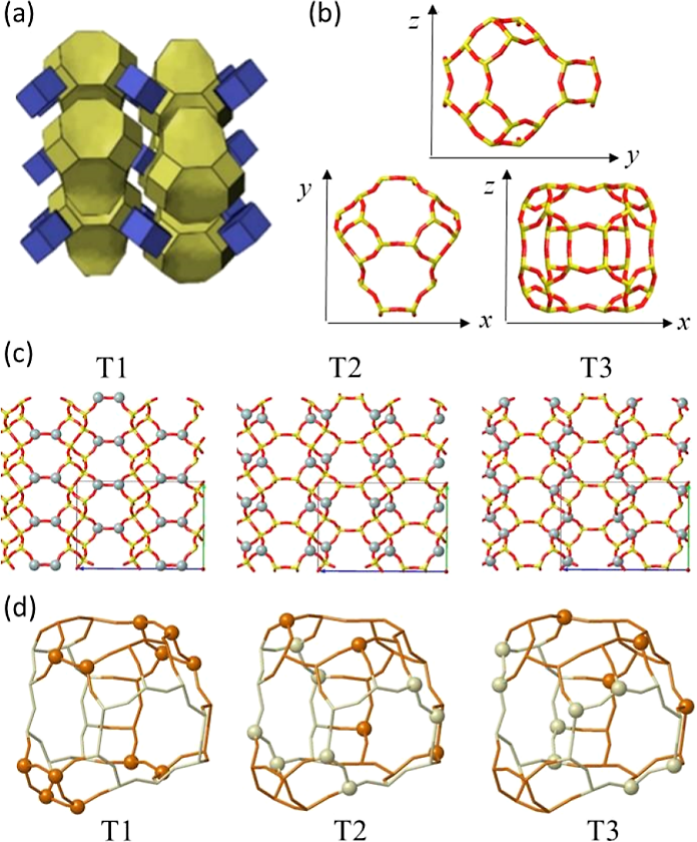
AEI 3D structure (a), cavity framework topology (b), T1, T2, and T3 sites in the AEI unit cell (c) and in the *t-per* or aei cavity (d). In (c), Si and O atoms are shown as yellow and red sticks, with specified T sites as gray balls. In (d), the specified T sites are shown as balls, with a color code indicating Al presence in T1 in an 8r window: brown for Al-T1-containing and gray for non-Al-T1-containing windows.

This work focuses on the H-SSZ-39 zeolite, an aluminosilicate with the AEI framework topology. The acid H-SSZ-39 zeolite shows excellent catalytic performance in some industrially relevant processes like the methanol-to-olefin (MTO) reaction,^49−53^ and the metal-exchanged Cu-SSZ-39 zeolite is used in environmental applications such as methane-to-methanol^53,54^ and exhaust gas treatment via the NH_3_-SCR-NOx reaction.^55−58^ The AEI crystallographic structure contains a three-dimensional pore system built up with *d6r* units arranged to form large asymmetrical pear-like cavities (labeled *t-per*) of 12.6 Å × 11.2 Å which are connected through 3.8 Å × 3.8 Å 8-membered ring (8r) windows (see Figure 1a,b).^59^ There are three different tetrahedral T-site positions in the AEI framework, T1, T2, and T3, and they are not equally distributed among the six 8r windows that give access to each cavity (Figure 1c,d). Thus, in each *t-per* or *aei* cavity, there are four 8r windows that contain the three T sites, 4 T1, 2 T2, and 2 T3 (brown windows in Figure 1d) and two 8r windows that contain 4 T2 and 4 T3 sites, so they lack T1 sites (gray windows in Figure 1d). Taking into account that the presence or absence of framework Al in the 8r windows of another cage-based zeolite, SSZ-13 with the CHA structure, has a great influence on the diffusion of olefins^60,61^ and on the NH_3_-SCR-NOx reaction rate,^62−64^ it appears that controlling the Al location in AEI may have important practical implications for zeolite catalysis and separation purposes.

The first successful synthesis of SSZ-39 zeolite was reported in 1999 by Zones et al. using cyclic or polycyclic quaternary ammonium cations as OSDAs.^65,66^ Since then, new synthetic routes have been developed to produce AEI zeolites with improved hydrothermal stability and catalytic performance, and nowadays SSZ-39 is obtained using a wide range of piperidinium-related OSDAs under a variety of inorganic synthesis conditions.^57,58,67−69^ However, the influence of OSDAs over Al siting in SSZ-39 remains unclear yet, and only a recent study by Xiao et al. shows that the number of Al pairs in the framework of SSZ-39 increases when the zeolite is synthesized with Na cations, without specifying which T sites are occupied.^54^

**Figure 2:**
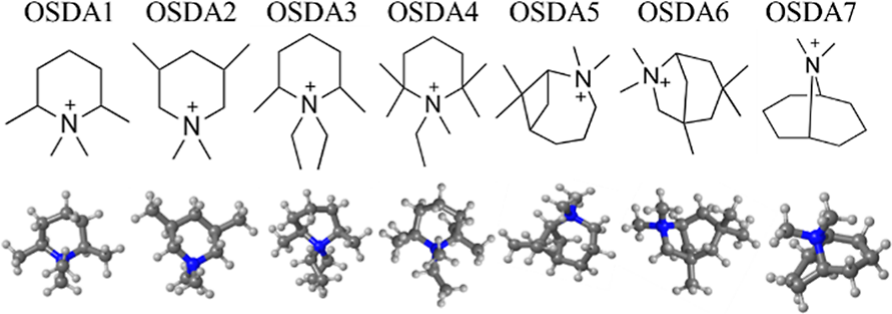
OSDAs studied. N, C, and H atoms depicted as blue, gray, and white balls, respectively.

In this work, we address the computational prediction of Al positioning in AEI using OSDAs with diverse molecular geometry and charge distribution (Figure 2). For this purpose, we apply AIMD simulations to investigate the mobility of conventional piperidinium-based OSDAs and unconventional azoniabicyclo- and azabicyclo-based OSDAs with different ring-substitutions (full chemical names are given in Table S1 in the Supporting Information) in pure silica and Al-containing AEI frameworks. The ultimate goal is to unravel whether the inclusion of both electrostatic and van der Waals interactions in the simulations leads to different mobilities and preferred orientations of some specific OSDAs inside the *aei* cavity and allows to predict their Al-directing ability.

## Computational Details

### Models

The SSZ-39 (AEI) structure was modeled by means of an orthorhombic unit cell (*Cmcm*) containing 48 Si and 96 O atoms with lattice parameters *a* = 13.448, *b* = 12.814 Å, *c* = 18.593 Å, and α = β = γ = 90° obtained after a static DFT full relaxation of atomic positions and unit cell parameters at the PBE-D3 level (see details in the Methods section). Next to the pure silica model with Si_48_O_96_ composition, three Al-containing models with only one Al atom per unit cell, that is, AlSi_47_O_96_ composition (Si/Al ratio = 47), were employed along the study. The three Al-containing models were generated by replacing a single-framework Si atom by Al in each of the three T1, T2, and T3 positions (Figure 1c). In the first set of static revPBE-D3 calculations, the AlO_4_^–^ negative charge was not compensated with any cation, thus providing the intrinsic relative stability of each isolated T site. These calculations show that T1 is the most stable position, followed by T2 and T3 that are 3 and 4 kJ/mol, respectively, less stable than T1.

### Methods

#### Static DFT

Periodic DFT calculations were performed with the Vienna Ab initio Simulation Package (VASP 5.4) code^70^ using the revPBE functional because of its improved performance for solid-state calculations.^71−73^ The valence density was expanded in a plane-wave basis set with a kinetic energy cutoff of 600 eV, and the effect of the core electrons in the valence density was taken into account by means of the projected augmented wave formalism.^74^ Integration in the reciprocal space was carried out at the Γ-point of the Brillouin zone. Dispersion corrections to the energies were evaluated using D3 Grimme’s method.^75,76^ Electronic energies were converged to 10^–6^ eV, and geometries were optimized until the forces on atoms were less than 0.015 eV/Å. During geometry optimizations, the positions of all atoms in the system were allowed to relax without any restriction while the unit cell parameters were kept constant. In order to avoid any artificial interactions caused by periodic images of OSDAs located in neighboring cavities, only one OSDA was introduced per AEI unit cell, leaving one empty cavity in *a* and *c* directions, where OSDAs in neighboring cages would be closer and connected through 8r windows, when the system is periodically replicated during the simulations. Interaction energies, *E*_int_, were calculated as
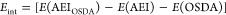
where *E*(AEI_OSDA_) is the total energy of the AEI zeolite framework containing one OSDA, *E*(AEI) is the total energy of the AEI unit cell, and *E*(OSDA) is the total energy of the isolated OSDA.

#### Molecular Dynamics

AIMD simulations were performed using the CP2K software package (CP2K 3.0).^77,78^ The AEI unit cell was optimized with an *NPT* stabilization run of 10 ps at *T* = 408 K, obtaining lattice parameters *a* = 13.752, *b* = 12.646 Å, *c* = 18.482 Å, and α = β = γ = 90°. Sampling of phase space occurs within the *NVT* ensemble using a time step of 0.5 fs for the integration of the equations of motion. The simulated temperature of 408 K was controlled by a Nosé–Hoover chain of five thermostat beads with a time constant of 100 wavenumbers.^79^ As a level of theory, the revPBE functional^71^ was chosen together with additional Grimme D3 dispersion corrections. A combination of a Gaussian (TZVP) and a plane wave basis set^73,80^ with a cutoff of 320 Ry was used, and GTH pseudopotentials were applied.^81,82^ The self-consistent field convergence criterion was set at 1 × 10^–6^ Ha. All systems were first equilibrated for 5 ps, followed by a production run of 100 ps when the Si_48_O_96_ model was used and 75 ps when the AlSi_47_O_96_ model was used. Longer simulations of 350 ps were performed for selected systems to confirm the validity of the simulation times chosen.

## Results and Discussion

### Mobility of Piperidinium-Based OSDAs Using Neutral Models

#### Influence of Size and Position of N- and Ring-Substituents

In this entire section, we investigate the van der Waals interactions between the neutral Si_48_O_96_ framework and neutral molecular models of the OSDAs, generated by replacing the N atom by a C atom. Note that throughout the section, the C atom substituting the original N in the cationic OSDA is labeled as “N” and is shown in blue in all figures.

*N*,*N*-Dimethyl-2,6-dimethyl (OSDA1), *N*,*N*-dimethyl-3,5-dimethyl (OSDA2), and *N*,*N*-diethyl-2,2,6,6-tetramethyl (OSDA3) piperidinium cations are the OSDAs most widely used nowadays to synthesize the SSZ-39 zeolite, and it has been claimed in the literature that the position of the methyl groups in the ring and their *cis* or *trans* conformation influence the synthesis and the final catalytic properties of the resulting material.^23,67^ The first part of the study analyzes (i) the influence of the position of the methyl ring-substituents, (ii) the size of the N-substituents (methyl or ethyl), and (iii) the size of the N-containing ring on the mobility of OSDAs inside the *aei* cage. For this purpose, regular AIMD simulations 100 ps long were performed for the *cis* and *trans* isomers of OSDA1, OSDA2, and OSDA3 inside the pure silica Si_48_O_96_ unit cell at a synthesis temperature of 408 K. In addition, a set of neutral versions of these three OSDAs, with ring sizes ranging from five carbon atoms (5-C) to ten carbon atoms (10-C) (see Figure S1), allowed the analysis of the effect of ring size.

**Figure 3:**
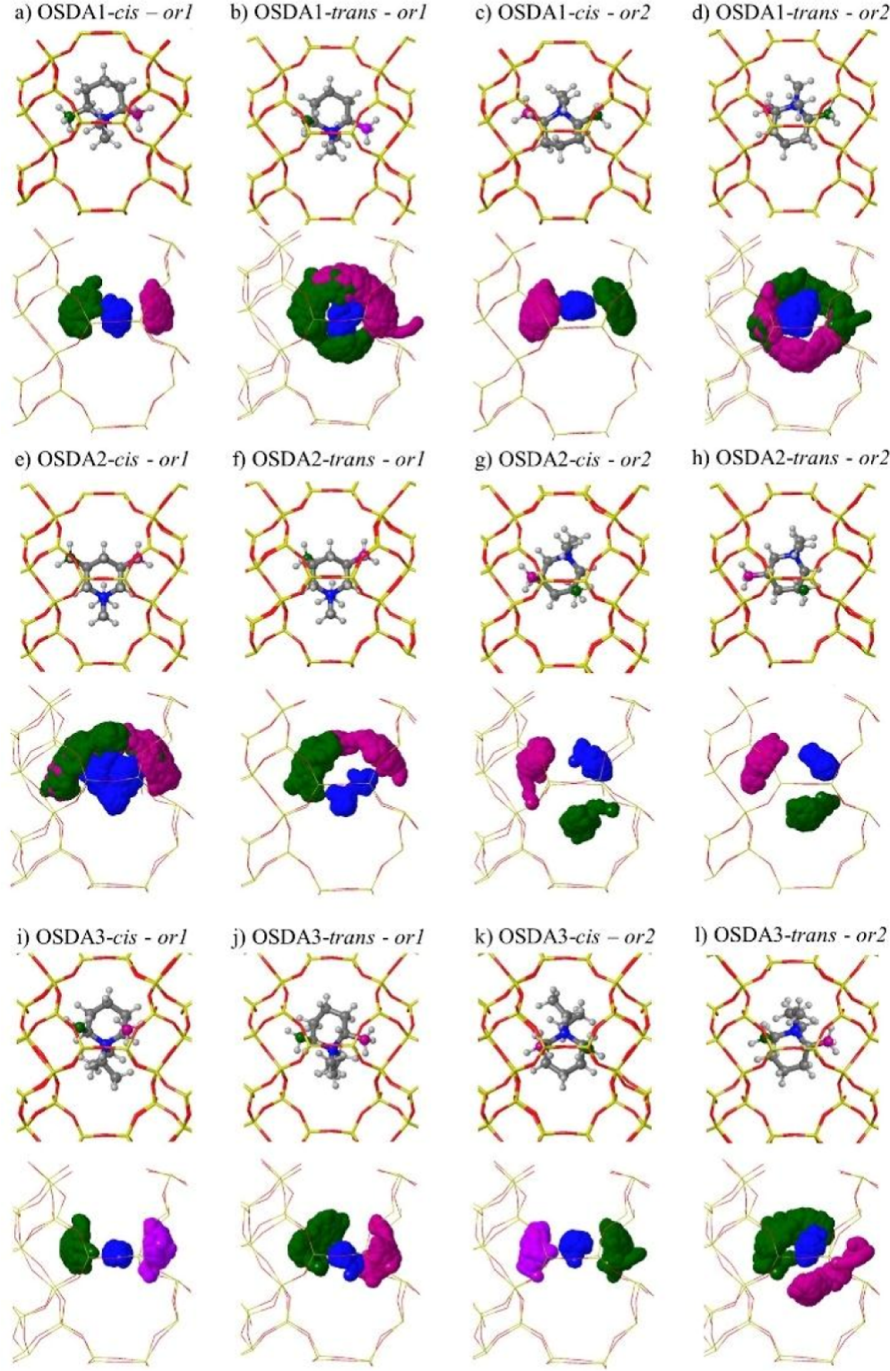
Initial geometries and scatterplots for the “N”- (blue) and methyl ring-substituents (green and purple) for OSDA1 (a–d), OSDA2 (e–h), and OSDA3 (i–l) obtained from 100 ps AIMD simulations at 408 K. Framework Si and O atoms depicted as yellow and red sticks. “N”, C, and H atoms depicted as blue, gray, and white balls.

**Figure 4:**
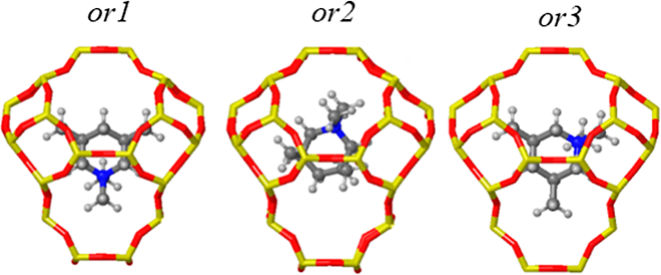
Possible orientations of OSDA2 within the *aei* cage.

Two initial orientations were chosen for each OSDA in the AIMD runs: one with the “N” atom facing the narrow part of the *aei* cage (*or1*) and the other facing the wide part of the cavity (*or2*), as shown in Figures 3 and 4. A general lack of mobility of the piperidinium-based OSDAs is observed in the scatterplots showing the position of the “N” atom (in blue) and of the two methyl substituents in the ring (in green and purple) during 100 ps of each simulation. A complete reorientation of the occluded OSDA from *or1* to *or2* or vice versa was never observed in any of the 12 simulations; the two methyl groups in the ring never swapped positions through full rotations, and the “N” atom movement was always restricted in a narrow region in the center of the cavity.

In some simulations, significant rotations of up to 10° within the *xy* plane, which is the molecular plane, were observed (Figure 3b,d,e), but in general these molecular rotations are smaller than 30°. For OSDA 2, a new orientation with the methyl substituents of the “N” atom pointing toward the wide side of the cavity, named *or3*, was found in the simulations starting from *or2* (see Figure 4). A 60° rotation in the *xy* plane occurred during the first 10 ps of the simulation for both the *cis* and *trans* isomers, and then the resulting *or3* orientation was maintained for the rest of the run because of the optimal fit of the methyl groups with the topology of the *aei* cavity (Figure 3g,h). The two ethyl groups attached to “N” in OSDA3 pose an additional steric hindrance that anchors the “N” atom in the center of the cavity, as illustrated by the blue scatterplots in Figure 3i–l and by the calculated rotation angles in the *xy* plane always smaller than 60°. “N” scatterplots for OSDA1 are similar to those of OSDA3 but with a slightly increased mobility along the *x* and *y* directions due to the smaller size of the methyl substituents at the N atom.

At this point, we assume that all possible stable orientations of the selected OSDAs inside *aei* cavities have been sampled, with just a new *or3* orientation appearing in the case of OSDA2 (see Figure 4). Therefore, all the piperidinium-based OSDAs show similar low mobility, with minor differences in the rotation within the *xy* plane due to the position of the methyl substituents. To quantify the relative stability of the OSDA orientations and their interaction energy (*E*_int_) with the AEI framework, static DFT calculations at the revPBE-D3 level were performed by using the initial models from the AIMD simulations. Table 1 shows that *or2* is disfavored for OSDA2 due to steric hindrance between the methyl substituents and the framework in the narrow part of the *aei* cavity, explaining the observed rotation from *or2* to *or3* in the simulations. The *E*_int_ values indicate that OSDA1 and OSDA3 can adopt any of the three orientations during SSZ-39 zeolite synthesis, although OSDA3 is more sensitive to orientation due to its larger N-substituents. In contrast, OSDA2 is predominantly found in *or1* and *or3*. On the other hand, the interaction energies normalized by the number of C atoms (*E*_int_/C) that allows the comparison of molecules of different sizes are similar, ∼−15 kJ/mol in agreement with the experimentally reported good structure-directing effect of all these piperidinium-based systems for AEI.^45,46,61−64^

**Table 1:** Absolute Interaction Energies *E*_int_ (in kJ/mol) and Interaction Energies Normalized per Number of C Atoms *E*_int_/C (in kJ/mol C) between Neutral OSDAs and the Neutral Si_48_O_96_ AEI Framework Obtained from Static revPBE-D3 Calculations

	*E*_int_ (kJ/mol)			*E*_int_/C (kJ/mol C)		
	*or1*	*or2*	*or3*	*or1*	*or2*	*or3*
OSDA1-*cis*	–151	–140	–151	–15	–14	–15
OSDA1-*trans*	–156	–159	–159	–16	–16	–16
OSDA2-*cis*	–151	–139	–152	–15	–14	–15
OSDA2-*trans*	–152	–138	–160	–15	–14	–16
OSDA3-*cis*	–184	–169	–188	–15	–14	–16
OSDA3-*trans*	–171	–197	–195	–14	–16	–16

#### Influence of Ring-Size

**Figure 5:**
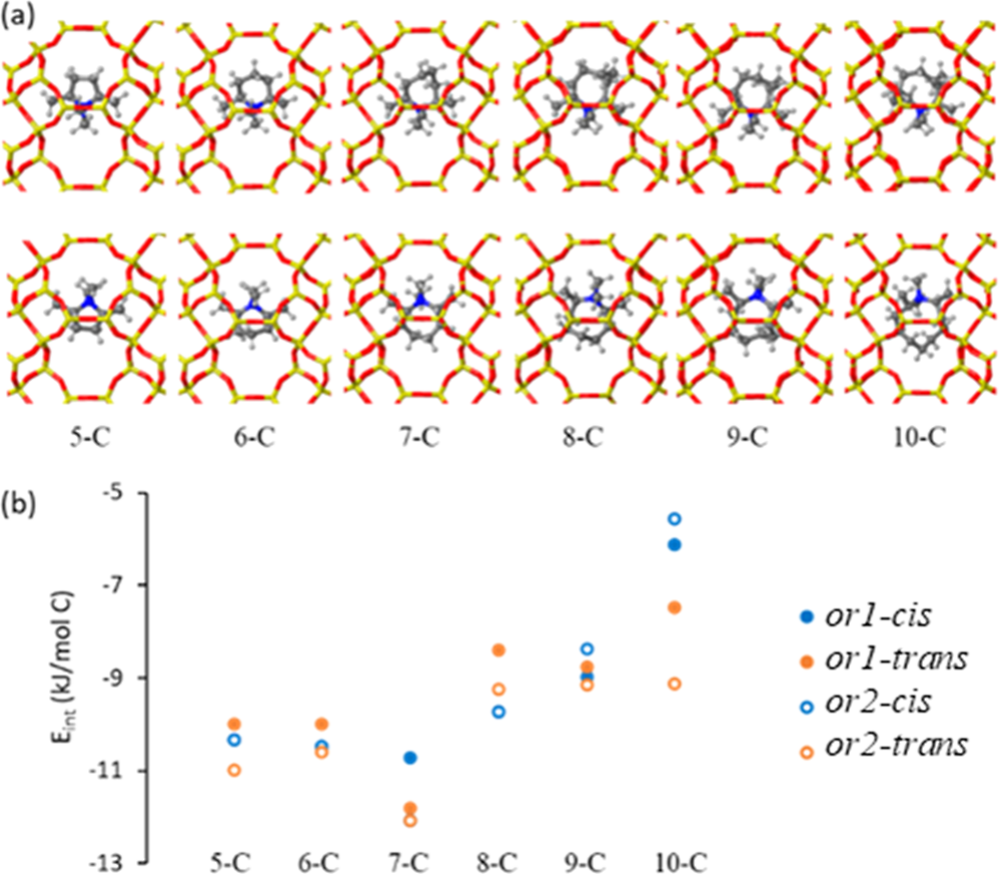
OSDA1 derivatives with ring sizes from 5-C to 10-C. (a) Optimized geometries of the *cis* isomers in *or1* (top) and *or2* (bottom) orientations inside the *aei* cavity and (b) normalized interaction energies *E*_int_/C (in kJ/mol C) for *cis* and *trans* isomers in *or1* and *or2* as a function of ring size. Framework Si and O atoms depicted as yellow and red sticks, and “N”, C, and H atoms depicted as blue, gray, and white balls, respectively.

The impact of ring size in cyclic OSDAs on their mobility and stability within the AEI framework was investigated here following the methodology employed in a previous section. Starting from OSDA1 with a six-carbon (6-C) ring, six neutral molecules with ring sizes ranging from five (5-C) to ten (10-C) carbon atoms were generated (Figures 5a and S1). Both *cis* and *trans* isomers of each molecule were placed in the *aei* cage in two orientations, *or1* and *or2*, and their interaction energies with the neutral Si_48_O_96_ AEI framework were calculated by using static PBE-D3 methods. The interaction energies (*E*_int_) and the normalized values per carbon atom (*E*_int_/C), summarized in Table S3 and Figure 5b, show that 7-C-OSDA1 fits best within the *aei* cavity, and increasing the ring size beyond seven carbon atoms is more destabilizing than reducing it to five or six. Piperidinium-type rings with eight or more carbon atoms do not fit well in the *aei* cages and may instead direct the synthesis toward frameworks with wider cavities. As a result, the AIMD study on OSDA mobility focused on the 5-C, 6-C, and 7-C versions of OSDA1, OSDA2, and OSDA3, with *E*_int_ and *E*_int_/C values provided in Table S4.

**Figure 6:**
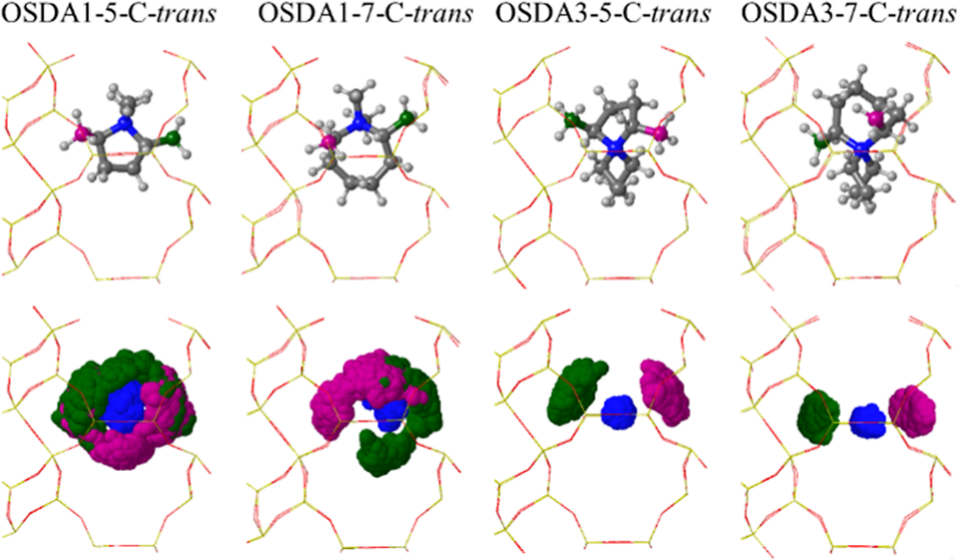
Initial geometries and scatterplots for the “N” (blue) and methyl ring-substituents (green and purple) for OSDA1 and OSDA3 with 5-C and 7-C ring sizes in *or2* orientation obtained from 100 ps AIMD simulations at 408 K. Framework Si and O atoms depicted as yellow and red sticks. “N”, C, and H atoms depicted as blue, gray, and white balls.

The scatterplots of the “N” atom position from 24 new AIMD simulations (Figures 6 and S2–S4) reveal that 5-C-ring molecules exhibit greater mobility for OSDA1 and OSDA2 compared to the 6-C versions, with rotations as large as 120° in some cases, while 7-C-ring systems show significantly reduced mobility, remaining mostly in their initial positions. OSDA3, however, remains immobile regardless of the ring size due to the strong blocking effect of the two ethyl substituents at the “N” atom, with in-plane rotations smaller than 20° (Figures 6 and S4). These qualitative and quantitative results suggest that the size of the N-substituents has a more significant impact on OSDA mobility than ring size within the 5-C to 7-C range. As the N-substituent size decreases from ethyl (OSDA3) to methyl (OSDA1), mobility increases across all ring sizes. For OSDA1 and OSDA2 with methyl substituents, increasing the ring size from 5-C to 7-C reduces the mobility slightly (Figure 6).

In summary, from the AIMD simulations and the static revPBE-D3 calculations performed with the neutral framework and neutral OSDA models that only take into account the van der Waals interactions between the occluded organic guest and the inorganic silicate host, it is concluded that all the piperidinium-based molecules considered with ring sizes between 5-C and 7-C are good OSDAs for the synthesis of the AEI framework. All of them can be stabilized within the *aei* cavity in two different orientations that never interconvert, and all of them have limited mobility within the *xy* plane that contains the molecule’s ring. This mobility is particularly hindered by the presence of ethyl substituents at the “N” atom and not so much by the ring size or the location of methyl substituents at the ring.

### Mobility of OSDAs and Al Distribution in AEI Zeolites Using Realistic Ionic Models

Once the low mobility of neutral models of piperidinium-based OSDAs in the neutral pure silica Si_48_O_96_ AEI framework was established, the dynamic behavior of the true cationic OSDAs in negatively charged Al-containing frameworks and the possibility to stabilize Al at specific T sites by means of enhanced electrostatic interactions with some particular OSDAs were explored next.

First, interaction energies (*E*_int_) between each of the seven cationic OSDAs (Figure 2) and the negatively charged AEI framework with one Al in the T1, T2, or T3 positions were estimated using static revPBE-D3 calculations, as summarized in Table 2. The Al atom was positioned at the T site closest to the OSDA’s N atom, and all OSDAs were placed in the three orientations (*or1*, *or2*, and *or3*) observed in the previous AIMD simulations. Only the *cis* configuration was considered for OSDA1, OSDA2, and OSDA3, due to the similarity with the *trans* conformers. Optimized geometries are shown in Figures S5–S11, and N–Al distances are listed in Tables 3 and S5.

**Table 2:**
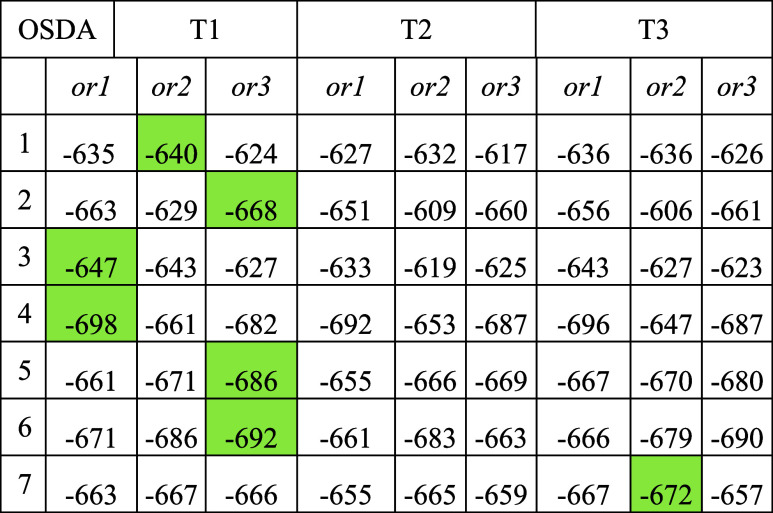
Interaction Energies *E*_int_ (in kJ/mol) between Cationic OSDAs and the Negatively Charged AlSi_47_O_96_ AEI Framework Obtained from Static revPBE-D3 Calculations^a^

a The lowest *E*_int_ value for each OSDA is highlighted.

**Table 3:** Relative Stability *E*_rel_ (in kJ/mol) of Al Position at T1, T2, and T3 in the Absence of a Compensating Cation and for Each OSDA Investigated in This Work and the Corresponding Optimized N–Al Distance (in Å), Obtained from Static revPBE-D3 Calculations^a^

	*E*_rel_ (kJ/mol)			*r*(N–Al) (Å)		
	T1	T2	T3	T1	T2	T3
	0	5	3			
OSDA1	0	8	5	4.9	5.8	5.4
OSDA2	0	8	7	**4.8**	5.6	5.0
OSDA3	0	19	7	**5.4**	6.9	5.2
OSDA4	0	7	2	5.0	6.1	5.2
OSDA5	0	17	6	4.8	5.5	4.9
OSDA6	0	29	2	4.8	5.7	5.4
OSDA7	2	9	0	5.0	5.7	5.6

a The most stable orientation of the OSDA inside the *aei* cavity is taken in all cases.

The large absolute *E*_int_ values, ranging from −606 to −698 kJ/mol (Table 2), reflect the strong electrostatic interactions between the negatively charged AlO_4_^–^ tetrahedra and positively charged OSDAs, along with van der Waals interactions between the organic species and the inorganic AEI framework (see Figure S12 in the Supporting Information). The presence of Al alters some trends found using neutral models, such as the relative stability of *or1*, *or2,* and *or3* orientations for OSDA1 and OSDA2. All orientations were equally stable in neutral models (see Table 1), but electrostatic interactions now create small differences for OSDA1, with *or3* becoming slightly destabilized compared to the others, and for OSDA2, with a significant destabilization of *or2* of around 40 kJ/mol (Table 2) due to a slight rotation in the *xy* plane (Figure S6). Generally, piperidinium-based OSDAs with 2,6-substitution, especially OSDA3 and OSDA4, favor the *or1* orientation, while OSDA2 and the bulkier OSDA5 and OSDA6 are more stable in the *or3* orientation across all T sites.

In the most stable structures for each OSDA, the cationic center at the N atom is typically close to the negatively charged AlO_4_^–^ unit, with N–Al distances shorter than 5 Å (see Tables 3 and S5 for N–Al distances and Tables 3 and S6 for relative stabilities). Conversely, less stable structures, such as OSDA2, OSDA5, and OSDA6 in T2-*or1* systems, have optimized N–Al distances as long as 7 Å. However, factors beyond electrostatic interactions play a role, as some stable structures exhibit N–Al distances larger than 5 Å (e.g., 5.4 Å for OSDA3 at T1-*or1*), while unstable systems can have similar N–Al distances of around 5 Å (e.g., OSDA4 T1-*or2* in Tables S5 and S6). As a result, no clear correlation was found between the relative *E*_int_ and N–Al distances (Figure S13).

The interaction energies for the cationic derivatives of OSDA1 and OSDA2 with 5-C and 7-C rings (Table S7) show that the structure-directing effect of 5-C rings is weaker, with *E*_int_ values averaging −646 and −648 kJ/mol for OSDA1–5-C and OSDA2–5-C, respectively, and stronger for 7-C rings, with *E*_int_ values of −669 and −663 kJ/mol for OSDA1–7-C and OSDA2–7-C, respectively. Again, this is not related with the optimized N–Al distances that are quite similar for the 5-C, 6-C, and 7-C systems (see Table S8).

**Figure 7:**
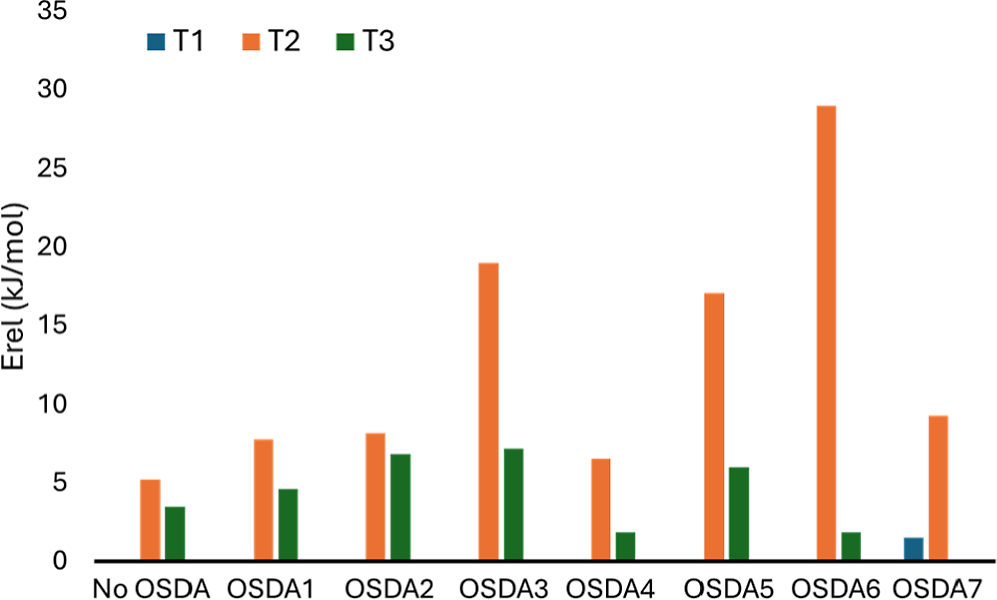
Relative revPBE-D3 stability *E*_rel_ (in kJ/mol) of Al position at T1, T2, and T3 in the absence of compensating cations and for each OSDA investigated in this work, taking in all cases the most stable OSDA orientation inside the *aei* cage.

Regarding Al positioning, T1 is the most stable site for all OSDAs except OSDA7 where T3 is preferred, while T2 is generally the least stable (Figure 7). The most stable system for OSDA7 is found with Al in T3, followed by T1. Given the inherent stability of Al-siting (T1 < T2 < T3 without charge-balancing species, as detailed in Computational Details), the relative energies in Table 3 and Figure 7 confirm that cationic OSDAs may influence Al stability at different T sites. However, the most stable structure for each OSDA does not always align with the shortest N–Al distance due to other attractive or repulsive interactions between the substituents and the framework. Overall, the revPBE-D3 results suggest that piperidinium-based OSDAs with the positive charge in a symmetric position in the ring and the two azoniabicylo-heptane or azepanium derivatives with the positive charge in a less symmetric position all favor Al placement at T1 in the AEI framework, with OSDA7 being the exception that is able to stabilize Al at T3.

**Figure 8:**
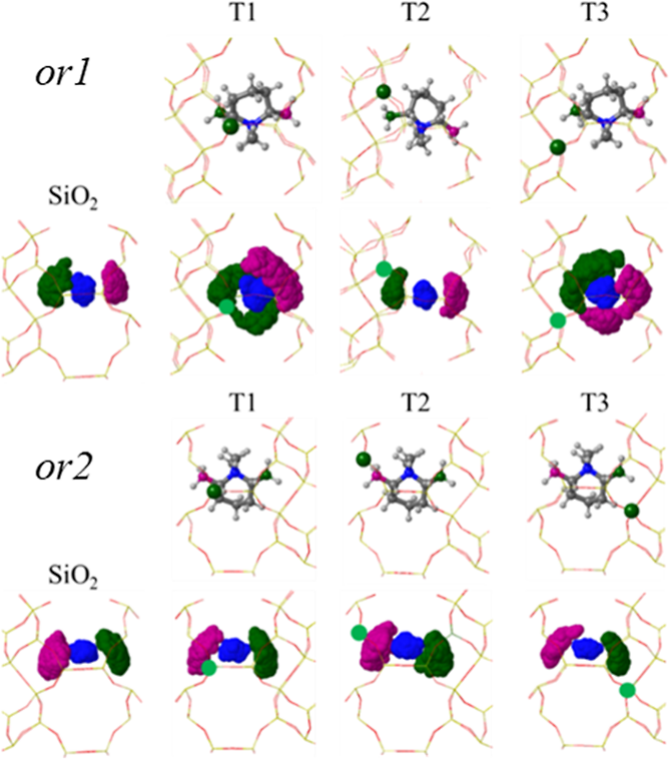
Initial geometries and scatterplots for the N atom (blue) and methyl ring-substituents (green and purple) for OSDA1 in an *aei* cavity containing one Al atom in each of the three T1, T2, and T3 positions, obtained from 75 ps AIMD simulations at 408 K. The scatterplots obtained with the neutral SiO_2_ model are included for comparison. Framework Si and O atoms depicted as yellow and red sticks. Al, N, C, and H atoms depicted as green, blue, gray, and white balls. For clarity, Al is depicted as light green circles in the scatterplots.

To improve the theoretical description of these systems under realistic conditions, regular AIMD simulations at 408 K were conducted for OSDA1, OSDA2, OSDA5, OSDA6, and OSDA7 in AlSi_47_O_96_ AEI models, with Al in the T1, T2, and T3 sites. OSDA3 and OSDA4 were excluded due to the immobilizing effect of ethyl substituents at the N atom found previously. Scatterplots of the N atom (blue) and two methyl C atoms (purple and green) during 75 ps of AIMD simulations for OSDA1 and OSDA2 (Figures 8 and S14) confirm the low mobility observed in the neutral models. The N atom consistently remains near its initial position due to the good fit of the molecules in the *aei* cavity, with small molecular rotations within the *xy* plane of less than 20° in most cases and only some occasional rotation of up to 100° as, for instance, *or1* with Al in T1 and T3 (Figure 8).

The location of T1 in the wider part of the *aei* cage (Figure 1c,d) facilitates the interaction with OSDAs that place their positive charge at the cavity center and show limited mobility. The average N–Al distances during the 75 ps simulations for OSDA1 and OSDA2 are only slightly longer than those from static revPBE-D3 calculations (Table S9).

**Figure 9:**
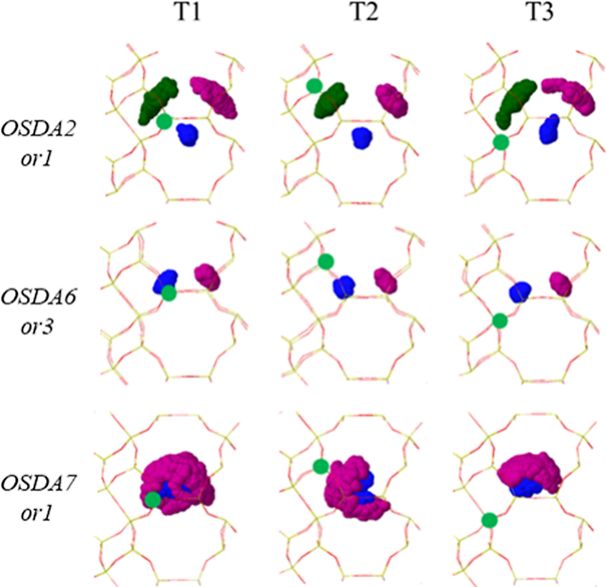
Scatterplots for the N atom (blue) and methyl ring-substituents (green and purple) for a piperidinium-based OSDA2, an azepanium-based OSDA6, and a nonsubstituted octane ring OSDA7 in an *aei* cavity containing one Al atom in each of the three T1, T2, and T3 positions, obtained from 75 ps AIMD simulations at 408 K. Framework Si and O atoms depicted as yellow and red sticks. Al, N, C, and H atoms depicted as green, blue, gray, and white balls.

For OSDA5 and OSDA6, only the most stable *or3* orientation was taken as the starting point for the AIMD simulations, which indicate a slightly lower mobility of these cations (Figures 9 and S15). On the one hand, the seven-membered rings are the best suited to fill the *aei* cavity as described before, and on the other hand, the ring substituents docked into the narrow part of the cavity restrict the rotations that can take place, clearly represented in the scatterplots of the C atoms plotted in purple. The asymmetric location of the N atom in these two OSDAs allows a closer interaction with the AlO_4_^–^ lattice centers at T3, with average N–Al distances as short as 4.5 Å for OSDA6 (see Table S9). However, this spatial proximity is not enough to reverse the preferred location of Al at the T1 site.

Unlike the similar behavior of the previously discussed OSDAs, ring substituents significantly affect the OSDA mobility. This is evident in the much higher mobility of the nonsubstituted octane ring (OSDA7) compared to the dimethyl-substituted hexane rings (OSDA1 and OSDA2). Figures 9 and S16 show full rotations of OSDA7, such as in *or1*-T1, where the purple scatterplot of the marked C atom forms a complete circle around the cavity, and the rotation within the *xy* plane covers all values between 0 and 180°. These rotations lead to interconversions between different orientations, regardless of Al placement. To verify OSDA7’s mobility, additional AIMD simulations were conducted starting from four different orientations (*or4* to *or7*), and the interaction energies (*E*_int_) obtained from static revPBE-D3 calculations, summarized in Table S9, confirm OSDA7’s consistent enhanced mobility due to the lack of ring substituents. The calculated *E*_int_ values remain within the range from −640 to −672 kJ/mol across all simulations.

The singularity of OSDA7 regarding its enhanced mobility is qualitatively observed in the scatterplots in Figure S16 and quantitatively reflected in the average N–Al distances. The average of the N–Al distance in the 75 ps AIMD simulations for each OSDA was compared with the corresponding N–Al distance obtained from the static revPBE-D3 calculations (see Table S9 and Figure S17). When the 42 data points are plotted together, a kind of linear trend is observed (Figure S17a) that clearly improves when the data corresponding to OSDA7 are removed from the set (Figure S17b). The correlation between dynamic and static N–Al distances is excellent for piperidinium-based and azepanium-based OSDAs, while for the 21 values corresponding to OSDA7, the correlation is clearly worse (Figure S17c).

The high mobility of OSDA7 necessitates a closer examination of the averaged N–Al distances from the AIMD simulations, as summarized in Table S10. When Al occupies T2, the N–Al distances are larger, ranging from 5.8 to 6.6 Å, similar to those of other OSDAs. In contrast, when Al is in T1 or T3, the distances shorten to 5.0–5.6 Å. Given OSDA7’s free rotation and orientation changes during the simulations, an average of all seven simulations provides a more accurate comparison. The average N–Al distances are 5.2 Å for T1, 6.3 Å for T2, and 5.2 Å for T3. The close proximity of OSDA7’s cationic center to T1 and T3, along with the larger interaction energy when Al occupies T3, suggests that OSDA7 could promote a different Al distribution in the AEI framework, favoring T3 over T1, unlike the other OSDAs that direct Al toward T1 sites.

**Figure 10:**
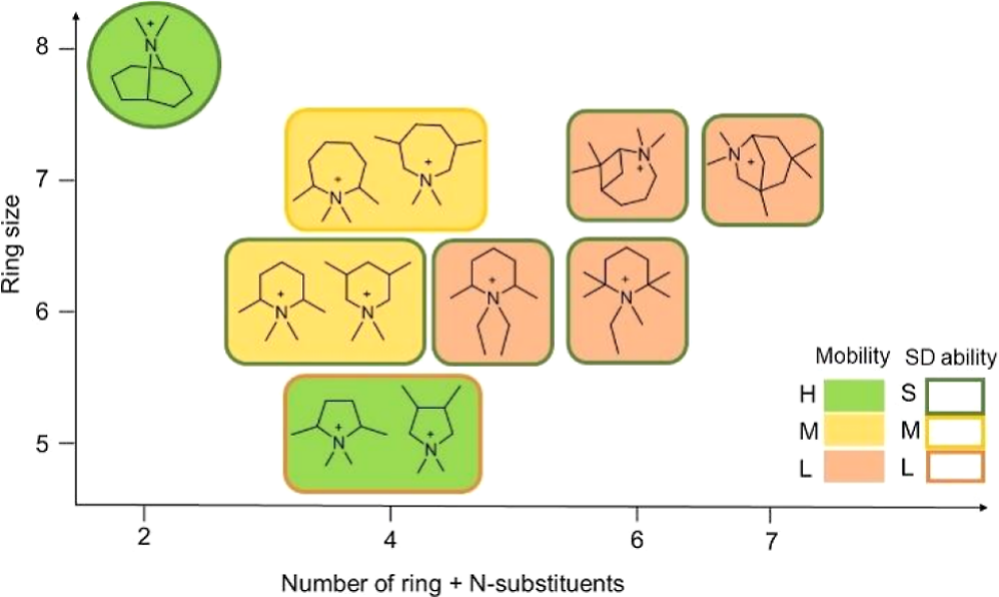
Overview of OSDAs’ geometrical structure, mobility, and structure-directing ability toward the AEI framework. Low (L), medium (M), and high (H) mobility are represented by orange, yellow, and green colors, respectively, while low (L), medium (M), and strong (S) SD ability are indicated by the solid lines framing the boxes with orange, yellow, and green colors, respectively. T1 is the most stable siting for Al, with the only exception of OSDA7 framed in a circle that stabilizes Al in T3.

In summary, the mobility of ring-based OSDAs is mostly determined by the number and size of ring and N substituents rather than by the ring size, as schematized in Figure 10. In contrast, the interaction energies between the OSDA and AEI framework are quite similar for all piperidinium-based and azepanium-based molecules considered, in agreement with their experimental ability to direct zeolite synthesis toward the AEI structure, and only OSDAs containing five-membered rings are too small to interact efficiently with the zeolite framework.

## Conclusions

In this study, DFT-based static calculations and AIMD simulations were employed to investigate the role of electrostatic interactions between cationic OSDAs and anionic AlO_4_^–^ centers in stabilizing Al at specific crystallographic positions within the AEI framework. The OSDAs analyzed include classical piperidinium-based molecules (OSDA1–OSDA4) with varying alkyl substituents in terms of size, conformation, and position, all with a symmetric charge distribution. Additionally, bulkier azepanium-based OSDAs with an asymmetric positive charge (OSDA5 and OSDA6) and an azabicyclo-octane-based OSDA with an unsubstituted cyclo-octane ring and external positive charge (OSDA7) were studied.

We first analyzed the contribution of van der Waals interactions to the stability and mobility of neutral piperidinium-based OSDAs in a pure silica AEI framework. Static revPBE-D3 calculations confirm a strong fit of all OSDAs within the AEI topology, while AIMD simulations reveal limited mobility, confining the N atom and its positive charge to a narrow region in the cavity center. Neither the *cis*/*trans* conformation nor the position of methyl ring-substituents (OSDA1/OSDA2) significantly affects mobility, and reducing the piperidinium ring size from 6 to 5 carbon atoms only slightly enhances movement within the *xy* plane. However, replacing methyl with an ethyl at the N atom (OSDA1/OSDA3) significantly restricts mobility regardless of the ring size, highlighting the strong influence of substituents on OSDA movement.

This critical role is confirmed in more realistic AIMD simulations with cationic OSDAs and Al-containing AEI zeolite models. Piperidinium-based (OSDA1–OSDA2) and azepanium-based (OSDA5–OSDA6) cations effectively fill the *aei* cage, preventing displacement from the cavity center with methyl substituents docking into the narrower parts of the cavity limiting possible rotations. In contrast, OSDA7, composed of an unsubstituted cyclo-octane ring, rotates freely and accesses regions of the cavity that are unreachable for the other OSDAs.

Static revPBE-D3 calculations suggest that all piperidinium-based OSDAs that place their positive charge at the center of the *aei* cavity preferentially stabilize Al at T1, the intrinsically most stable site. Although azepanium-based OSDAs (OSDA5 and OSDA6) can shift the positive charge away from the cavity center, this does not alter the intrinsic stability order of the T sites, with Al still favored at T1, closely followed by T3 for OSDA6. In contrast, OSDA7’s high mobility and ability to access narrow regions of the cavity might result in a different Al distribution, with T3 being the most stable site, followed by T1.

In conclusion, this study has identified structural features of cyclic OSDAs for AEI synthesis that increase the likelihood of placing Al at T sites other than the most stable T1, leading to an AEI catalyst distinct from those synthesized with conventional piperidinium-based OSDAs (OSDA1–OSDA4). When AEI synthesis is performed with the highly mobile OSDA7, which allows the positive charge to explore all positions within the cavity, an AEI catalyst with enhanced diffusion of short-chain olefins or NH_3_-SCR species may result, as more 8r windows accessing the cavity might contain a framework Al atom.

## Supporting Information Available

Optimized geometries, absolute interaction energies, and relative stability of OSDAs within the AEI crystallographic structure from static PBE-D3 calculations; scatterplots of N and C movement inside the AEI cavities from AIMD simulations at 408 K; and correlation between static and dynamic averaged N–Al distances (PDF)

## References

[ref1] Vermeiren W.; Gilson J. P. Impact of zeolites on the petroleum and petrochemical industry. Top. Catal. 2009, 52, 1131–1161. 10.1007/s11244-009-9271-8.

[ref2] Li Y.; Li L.; Yu J. Applications of zeolites in sustainable chemistry. Chem 2017, 3, 928–949. 10.1016/j.chempr.2017.10.009.

[ref3] Dusselier M.; Davis M. E. Small-pore zeolites: synthesis and catalysis. Chem. Rev. 2018, 118, 5265–5329. 10.1021/acs.chemrev.7b00738. 29746122

[ref4] Del Campo P.; Martínez C.; Corma A. Activation and conversion of alkanes in the confined space of zeolite-type materials. Chem. Soc. Rev. 2021, 50, 8511–8595. 10.1039/D0CS01459A. 34128513

[ref5] Cnudde P.; Waroquier M.; Van Speybroeck V. Universal descriptors for zeolite topology and acidity to predict the stability of butene cracking intermediates. Catal. Sci. Technol. 2023, 13, 4857–4872. 10.1039/D3CY00642E.

[ref6] Moliner M.; Boronat M. Towards enzyme-like zeolite designs to maximize the efficiency of catalysts by molecular recognition: fine-tuning confinement and active site location. Microporous Mesoporous Mater. 2023, 358, 112354 10.1016/j.micromeso.2022.112354.

[ref7] Shantz D. F.; Fild C.; Koller H.; Lobo R. F. Guest–host interactions in as-made Al-SM-12: implications for the synthesis of zeolite catalysts. J. Phys. Chem. B 1999, 103, 10858–10865. 10.1021/jp992549u.

[ref8] Pinar A. B.; Gómez-Hortigüela L.; McCusker L. B.; Pérez-Pariente J. Controlling the aluminum distribution in the zeolite ferrierite via the organic structure directing agent. Chem. Mater. 2013, 25, 3654–3661. 10.1021/cm4018024.

[ref9] Muraoka K.; Chaikittisilp W.; Yanaba Y.; Yoshikawa T.; Okubo T. Directing aluminum atoms into energetically favorable tetrahedral sites in a zeolite framework by using organic structure-directing agents. Angew. Chem., Int. Ed. 2018, 57, 3742–3746. 10.1002/anie.201713308. 29405535

[ref10] Moliner M.; Rey F.; Corma A. Towards the rational design of efficient organic structure-directing agents for zeolite synthesis. Angew. Chem., Int. Ed. 2013, 52, 13880–13889. 10.1002/anie.201304713. 24115577

[ref11] Gallego E. M.; Paris C.; Díaz-Rey M. R.; Martínez-Armero M. E.; Martínez-Triguero J.; Martínez C.; Moliner M.; Corma A. Simple organic structure-directing agents for synthesizing nanocrystalline zeolites. Chem. Sci. 2017, 8, 8138–8149. 10.1039/C7SC02858J. 29568462 PMC5855293

[ref12] Li Y.; Cao H.; Yu J. Toward a new era of designed synthesis of nanoporous zeolitic materials. ACS Nano 2018, 12, 4096–4104. 10.1021/acsnano.8b02625. 29714474

[ref13] Li J.; Gao M.; Yan W.; Yu J. Regulation of the Si/Al ratios and Al distributions of zeolites and their impact on properties. Chem. Sci. 2023, 14, 1935–1959. 10.1039/D2SC06010H. 36845940 PMC9945477

[ref14] Chizallet C. Toward the atomic scale simulation of intricate acidic aluminosilicate catalysts. ACS Catal. 2020, 10, 5579–5601. 10.1021/acscatal.0c01136.

[ref15] Van Speybroeck V.; Bocus M.; Cnudde P.; Vanduyfhuys L. Operando modeling of zeolite-catalyzed reactions using first-principles molecular dynamics simulations. ACS Catal. 2023, 13, 11455–11493. 10.1021/acscatal.3c01945. 37671178 PMC10476167

[ref16] Gallego E. M.; Portilla M. T.; Paris C.; Leon-Escamilla A.; Boronat M.; Moliner M.; Corma A. Ab initio synthesis of zeolites for preestablished catalytic reactions. Science 2017, 355, 1051–1054. 10.1126/science.aal0121. 28280200

[ref17] Ferri P.; Li C.; Schwalbe-Koda D.; Xie M.; Moliner M.; Gomez-Bombarelli R.; Boronat M.; Corma A. Approaching enzymatic catalysis with zeolites or how to select one reaction mechanism competing with others. Nat. Commun. 2023, 14, 2878 10.1038/s41467-023-38544-z. 37208318 PMC10198988

[ref18] Chien S. C.; Auerbach S. M.; Monson P. A. Modeling the self-assembly of silica-templated nanoparticles in the initial stages of zeolite formation. Langmuir 2015, 31, 4940–4949. 10.1021/acs.langmuir.5b00382. 25872102

[ref19] Khan M. N.; Auerbach S. M.; Monson P. A.; Monson P. A. Lattice Monte Carlo simulations in search of zeolite analogues: effects of structure directing agents. J. Phys. Chem. C 2015, 119, 28046–28054. 10.1021/acs.jpcc.5b09450.

[ref20] Bores C.; Auerbach S. M.; Monson P. A. Modeling the role of excluded volume in zeolite structure direction. J. Phys. Chem. Lett. 2018, 9, 3703–3707. 10.1021/acs.jpclett.8b01467. 29909633

[ref21] Lewis D. W.; Willock D. J.; Catlow C. R. A.; Thomas J. M.; Hutchings G. J. De novo design of structure-directing agents for the synthesis of microporous solids. Nature 1996, 382, 604–606. 10.1038/382604a0.

[ref22] Pophale R.; Daeyaert F.; Deem M. W. Computational prediction of chemically synthesizable organic structure-directing agents for zeolites. J. Mater. Chem. A 2013, 1, 6750–6760. 10.1039/c3ta10626h.

[ref23] Muraoka K.; Chaikittisilp W.; Okubo T. Multi-objective molecular design of organic structure-directing agents for zeolites using nature-inspired ant colony optimization. Chem. Sci. 2020, 11, 8214–8223. 10.1039/D0SC03075A. 34094176 PMC8163217

[ref24] Waitt C.; Gao X.; Gounder R.; Debellis A.; Prasad S.; Moini A.; Schneider W. F. Analysis on the guest/host relationships in the synthesis of the novel cage-based zeolites SSZ-35, SSZ-36, and SSZ-39. J. Phys. Chem. C 2023, 127, 22740–22751. 10.1021/acs.jpcc.3c05421.

[ref25] Schmidt J. E.; Deem M. W.; Davis M. E. Synthesis of a specified silica molecular sieve by using computationally predicted organic structure-directing agents. Angew. Chem., Int. Ed. 2014, 53, 8372–8374. 10.1002/anie.201404076. 24961789

[ref26] Daeyaert F.; Deem M. W. In silico design of chiral dimers to direct the synthesis of a chiral zeolite. Mol. Phys. 2018, 116, 2836–2855. 10.1080/00268976.2018.1492747.

[ref27] Waitt C.; Gao X.; Gounder R.; Debellis A.; Prasad S.; Moini A.; Schneider W. F. Analysis and augmentation of guest–host interaction energy models as CHA and AEI zeolite crystallization phase predictors. J. Phys. Chem. C 2023, 127, 22740–22751. 10.1021/acs.jpcc.3c05421.

[ref28] Li C.; Paris C.; Martinez-Triguero J.; Boronat M.; Moliner M.; Corma A. Synthesis of reaction-adapted zeolites as methanol-to-olefins catalysts with mimics of reaction intermediates as organic structure-directing agents. Nat. Catal. 2018, 1, 547–554. 10.1038/s41929-018-0104-7.

[ref29] Daeyaert F.; Ye F.; Deem M. W. Machine-learning approach to the design of OSDAs for zeolite beta. Proc. Natl. Acad. Sci. U.S.A. 2019, 116, 3413–3418. 10.1073/pnas.1818763116. 30733290 PMC6397530

[ref30] Schwalbe-Koda D.; Kwon S.; Paris C.; Bello-Jurado E.; Jensen Z.; Olivetti E.; Willhammar T.; Corma A.; Roman-Leshkov Y.; Moliner M.; Gomez-Bombarelli R. A priori control of zeolite phase competition and intergrowth with high-throughput simulations. Science 2021, 374, 308–315. 10.1126/science.abh3350. 34529493

[ref31] Sklenak S.; Dědeček J.; Li C.; Wichterlová B.; Gábová V.; Sierka M.; Sauer J. Aluminum siting in silicon-rich zeolite frameworks: a combined high-resolution ^27^Al NMR spectroscopy and quantum mechanics/molecular mechanics study of ZSM-5. Angew. Chem., Int. Ed. 2007, 46, 7286–7289. 10.1002/anie.200702628. 17768742

[ref32] Di Iorio J. R.; Gounder R. Controlling the isolation and pairing of aluminum in chabazite zeolites using mixtures of organic and inorganic structure-directing agents. Chem. Mater. 2016, 28, 2236–2247. 10.1021/acs.chemmater.6b00181.

[ref33] Zhao Z.; Xing Y.; Li S.; Meng X.; Xiao F.; McGuire R.; Parvulescu A.; Müller U.; Zhang W. Mapping Al distributions in SSZ-13 zeolites from ^23^Na solid-state NMR spectroscopy and DFT calculations. J. Phys. Chem. C 2018, 122, 9973–9979. 10.1021/acs.jpcc.8b01423.

[ref34] Knott B. C.; Nimlos C. T.; Robichaud D. J.; Nimlos M. R.; Kim S.; Gounder R. Consideration of the aluminum distribution in zeolites in theoretical and experimental catalysis research. ACS Catal. 2018, 8, 770–784. 10.1021/acscatal.7b03676.

[ref35] Nimlos C. T.; Hoffman A. J.; Hur Y. G.; Lee B. J.; Di Iorio J. R.; Hibbitts D. D.; Gounder R. Experimental and theoretical assessments of aluminum proximity in MFI zeolites and its alteration by organic and inorganic structure-directing agents. Chem. Mater. 2020, 32, 9277–9298. 10.1021/acs.chemmater.0c03154.

[ref36] Di Iorio J. R.; Li S.; Jones C. B.; Nimlos C. T.; Wang Y.; Kunkes E.; Vattipalli V.; Prasad S.; Moini A.; Schneider W. F.; Gounder R. Cooperative and competitive occlusion of organic and inorganic structure-directing agents within chabazite zeolites influences their aluminum arrangement. J. Am. Chem. Soc. 2020, 142, 4807–4819. 10.1021/jacs.9b13817. 32053365

[ref37] Wang Z.; Chu W.; Zhao Z.; Liu Z.; Chen H.; Xiao D.; Gong K.; Li F.; Li X.; Hou G. The role of organic and inorganic structure-directing agents in selective Al substitution of zeolite. J. Phys. Chem. Lett. 2021, 12, 9398–9406. 10.1021/acs.jpclett.1c01448. 34553943

[ref38] Bickel E. E.; Hoffman A. J.; Lee S.; Snider H. E.; Nimlos C. T.; Zamiechowski N. K.; Hibbitts D. D.; Gounder R. Altering the arrangement of framework Al atoms in MEL zeolites using mixtures of tetrabutylammonium and sodium structure-directing agents. Chem. Mater. 2022, 34, 6835–6852. 10.1021/acs.chemmater.2c01083.

[ref39] Bhan A.; Allian A. D.; Sunley G. J.; Law D. J.; Iglesia E. Specificity of sites within eight-membered ring zeolite channels for carbonylation of methyls to acetyls. J. Am. Chem. Soc. 2007, 129, 4919–4924. 10.1021/ja070094d. 17397162

[ref40] Boronat M.; Martínez-Sánchez C.; Law D. J.; Corma A. Enzyme-like specificity in zeolites: a unique site position in mordenite for selective carbonylation of methanol and dimethyl ether with CO. J. Am. Chem. Soc. 2008, 130, 16316–16323. 10.1021/ja805607m. 18986144

[ref41] Yokoi T.; Mochizuki H.; Namba S.; Kondo J. N.; Tatsumi T. Control of the Al distribution in the framework of ZSM-5 zeolite and its evaluation by solid-state NMR technique and catalytic properties. J. Phys. Chem. C 2015, 119, 15303–15315. 10.1021/acs.jpcc.5b03289.

[ref42] Gallego E. M.; Li C.; Paris C.; Martin N.; Martinez-Triguero J.; Boronat M.; Moliner M.; Corma A. Making nanosized CHA zeolites with controlled Al distribution for optimizing methanol-to-olefin performance. Chem.—Eur. J. 2018, 24, 14631–14635. 10.1002/chem.201803637. 30070401

[ref43] Li C.; Vidal-Moya A.; Miguel P. J.; Dědeček J.; Boronat M.; Corma A. Selective introduction of acid sites in different confined positions in ZSM-5 and its catalytic implications. ACS Catal. 2018, 8, 7688–7697. 10.1021/acscatal.8b02112.

[ref44] Zhang J.; Shan Y.; Zhang L.; Du J.; He H.; Han S.; Lei C.; Wang S.; Fan W.; Feng Z.; Liu X.; Meng X.; Xiao F. Importance of controllable Al sites in CHA framework by crystallization pathways for NH_3_-SCR reaction. Appl. Catal., B 2020, 277, 119193 10.1016/j.apcatb.2020.119193.

[ref45] Tang X.; Liu Z.; Huang L.; Chen W.; Li C.; Wang G.; Li G.; Yi X.; Zheng A. Violation or abidance of Löwensteins rule in zeolites under synthesis conditions?. ACS Catal. 2019, 9, 10618–10625. 10.1021/acscatal.9b01844.

[ref46] Tang X.; Chen W.; Dong W.; Liu Z.; Yuan J.; Xia H.; Yi X.; Zheng A. Framework aluminum distribution in ZSM-5 zeolite directed by organic structure-directing agents: a theoretical investigation. Catal. Today 2022, 405–406, 101–110. 10.1016/j.cattod.2022.06.027.

[ref47] Lee S.; Nimlos C. T.; Kipp E. R.; Wang Y.; Gao X.; Schneider W. F.; Lusardi M.; Vattipalli V.; Prasad S.; Moini A.; Gounder R. Evolution of framework Al arrangements in CHA zeolites during crystallization in the presence of organic and inorganic structure-directing agents. Cryst. Growth Des. 2022, 22, 6275–6295. 10.1021/acs.cgd.2c00856.

[ref48] Wang X.; Wang Y.; Moini A.; Gounder R.; Maginn E. J.; Schneider W. F. Influence of an N,N,N-trimethyl-1-adamantyl ammonium (TMAda^+^) structure directing agent on Al distributions and pair features in chabazite zeolite. Chem. Mater. 2022, 34, 10811–10822. 10.1021/acs.chemmater.2c01465.

[ref49] Dusselier M.; Deimund M. A.; Schmidt J. E.; Davis M. E. Methanol-to-olefins catalysis with hydrothermally treated zeolite SSZ-39. ACS Catal. 2015, 5, 6078–6085. 10.1021/acscatal.5b01577.

[ref50] Martín N.; Li Z.; Martinez-Triguero J.; Yu J.; Moliner M.; Corma A. Nanocrystalline SSZ-39 zeolite as an efficient catalyst for the methanol-to-olefin (MTO) process. Chem. Commun. 2016, 52, 6072–6075. 10.1039/C5CC09719C. 26947336

[ref51] Kang J. H.; Alshafei F. H.; Zones S. I.; Davis M. E. Cage-defining ring: a molecular sieve structural indicator for light olefin product distribution from the methanol-to-olefins reaction. ACS Catal. 2019, 9, 6012–6019. 10.1021/acscatal.9b00746.

[ref52] Ferri P.; Li C.; Paris C.; Vidal-Moya A.; Moliner M.; Boronat M.; Corma A. Chemical and structural parameter connecting cavity architecture, confined hydrocarbon pool species, and MTO product selectivity in small-pore cage-based zeolites. ACS Catal. 2019, 9, 11542–11551. 10.1021/acscatal.9b04588.

[ref53] Mahyuddin M. H.; Staykov A.; Shiota Y.; Miyanishi M.; Yoshizawa K. Roles of zeolite confinement and Cu–O–Cu angle on the direct conversion of methane to methanol by [Cu_2_(μ-O)]^2+^-exchanged AEI, CHA, AFX, and MFI zeolites. ACS Catal. 2017, 7, 3741–3751. 10.1021/acscatal.7b00588.

[ref54] Xiao P.; Wang Y.; Lu Y.; De Baerdemaeker T.; Parvulescu A.; Müller U.; De Vos D.; Meng X.; Xiao F.; Zhang W.; Marler B.; Kolb U.; Gies H.; Yokoi T. Effects of Al distribution in the Cu-exchanged AEI zeolites on the reaction performance of continuous direct conversion of methane to methanol. Appl. Catal., B 2023, 325, 122395 10.1016/j.apcatb.2023.122395.

[ref55] Moliner M.; Franch C.; Palomares E.; Grill M.; Corma A. Cu–SSZ-39, an active and hydrothermally stable catalyst for the selective catalytic reduction of NO_x_. Chem. Commun. 2012, 48, 8264–8266. 10.1039/c2cc33992g. 22782014

[ref56] Sonoda T.; Maruo T.; Yamasaki Y.; Tsunoji N.; Takamitsu Y.; Sadakane M.; Sano T. Synthesis of high-silica AEI zeolites with enhanced thermal stability by hydrothermal conversion of FAU zeolites, and their activity in the selective catalytic reduction of NO_x_ with NH_3_. J. Mater. Chem. A 2015, 3, 857–865. 10.1039/C4TA05621C.

[ref57] Martín N.; Boruntea C. R.; Moliner M.; Corma A. Efficient synthesis of the Cu-SSZ-39 catalyst for DeNO_x_ applications. Chem. Commun. 2015, 51, 11030–11033. 10.1039/C5CC03200H. 26074277

[ref58] Xu H.; Zhang J.; Wu Q.; Chen W.; Lei C.; Zhu Q.; Han S.; Fei J.; Zheng A.; Zhu L.; Meng X.; Maurer S.; Dai D.; Parvulescu A.; Müller U.; Xiao F. Direct synthesis of aluminosilicate SSZ-39 zeolite using colloidal silica as a starting source. ACS Appl. Mater. Interfaces 2019, 11, 23112–23117. 10.1021/acsami.9b03048. 31252486

[ref59] Baerlocher C.; Brouwer D.; Marler B.; McCusker L. B. Database of Zeolite Structures. https://www.iza-structure.org/databases/.

[ref60] Cnudde P.; Redekop E. A.; Dai W.; Porcaro N. G.; Waroquier M.; Bordiga S.; Hunger M.; Li L.; Olsbye U.; Van Speybroeck V. Experimental and theoretical evidence for the promotional effect of acid sites on the diffusion of alkenes through small-pore zeolites. Angew. Chem., Int. Ed. 2021, 60, 10016 10.1002/anie.202017025. PMC8251642 33496374

[ref61] Cnudde P.; Demuynck R.; Vandenbrande S.; Waroquier M.; Sastre G.; Speybroeck V. V. Light olefin diffusion during the MTO process on H-SAPO-34: a complex interplay of molecular factors. J. Am. Chem. Soc. 2020, 142, 6007–6017. 10.1021/jacs.9b10249. 32157875

[ref62] Krishna S. H.; Goswami A.; Wang Y.; Jones C. B.; Dean D. P.; Miller J. T.; Schneider W. F.; Gounder R. Influence of framework Al density in chabazite zeolites on copper ion mobility and reactivity during NO_x_ selective catalytic reduction with NH_3_. Nat. Catal. 2023, 6, 276–285. 10.1038/s41929-023-00932-5.

[ref63] Millan R.; Bello-Jurado E.; Moliner M.; Boronat M.; Gomez-Bombarelli R. Effect of framework composition and NH_3_ on the diffusion of Cu in Cu-CHA catalysts predicted by machine-learning accelerated molecular dynamics. ACS Cent. Sci. 2023, 9, 2044–2056. 10.1021/acscentsci.3c00870. 38033797 PMC10683499

[ref64] DeLuca M.; Jones C. B.; Krishna S. H.; Goswami A.; Saxena R.; Li S.; Prasad S.; Moini A.; Schneider W. F.; Gounder R. Effects of zeolite framework topology on Cu(I) oxidation and Cu(II) reduction kinetics of NO_x_ selective catalytic reduction with NH_3_. Chem Catal. 2023, 3, 100726 10.1016/j.checat.2023.100726.

[ref65] Zones S. I.; Nakagawa Y.; Evans S. T.; Lee G. S. Zeolite SSZ-39. US595837OA Patent, 1999.

[ref66] Wagner P.; Nakagawa Y.; Lee G. S.; Davis M. E.; Elomari S.; Medrud R. C.; Zones S. I. Guest/host relationships in the synthesis of the novel cage-based zeolites SSZ-35, SSZ-36, and SSZ-39. J. Am. Chem. Soc. 2000, 122, 263–273. 10.1021/ja990722u.

[ref67] Dusselier M.; Schmidt J. E.; Moulton R.; Haymore B.; Hellums M.; Davis M. E. Influence of organic structure-directing agent isomer distribution on the synthesis of SSZ-39. Chem. Mater. 2015, 27, 2695–2702. 10.1021/acs.chemmater.5b00651.

[ref68] Schmidt J. E.; Deem M. W.; Lew C.; Davis T. M. Computationally-guided synthesis of the 8-ring zeolite AEI. Top. Catal. 2015, 58, 410–415. 10.1007/s11244-015-0381-1.

[ref69] Hu P.; Iyoki K.; Fujinuma H.; Yu J.; Yu S.; Anand C.; Yanaba Y.; Okubo T.; Wakihara T. Broadening synthetic scope of SSZ-39 zeolite for NH_3_-SCR: a fast and direct route from amorphous starting materials. Microporous Mesoporous Mater. 2022, 330, 111583 10.1016/j.micromeso.2021.111583.

[ref70] Kresse G.; Furthmüller J. Efficient iterative schemes for ab initio total-energy calculations using a plane-wave basis set. Phys. Rev. B:Condens. Matter Mater. Phys. 1996, 54, 11169–11186. 10.1103/PhysRevB.54.11169. 9984901

[ref71] Zhang Y.; Yang W. Comment on Generalized Gradient Approximation Made Simple. Phys. Rev. Lett. 1998, 80, 890 10.1103/PhysRevLett.80.890.

[ref72] Yang K.; Zheng J.; Zhao Y.; Truhlar D. G. Tests of the RPBE, revPBE, HCTHhyb, and MOHLYP density functional approximations and 29 others against representative databases for diverse bond energies and barrier heights in catalysis. J. Chem. Phys. 2010, 132, 164117 10.1063/1.3382342. 20441268

[ref73] Lippert G.; Hutter J.; Parrinello M. A hybrid Gaussian and plane wave density functional scheme. Mol. Phys. 1997, 92, 477–487. 10.1080/00268979709482119.

[ref74] Blöchl P. E. Projector augmented-wave method. Phys. Rev. B:Condens. Matter Mater. Phys. 1994, 50, 17953 10.1103/PhysRevB.50.17953. 9976227

[ref75] Grimme S. Accurate description of van der Waals complexes by density functional theory including empirical corrections. J. Comput. Chem. 2004, 25, 1463–1473. 10.1002/jcc.20078. 15224390

[ref76] Grimme S.; Antony J.; Ehrlich S.; Krieg H. A consistent and accurate ab initio parametrization of density functional dispersion correction (DFT-D) for the 94 elements H–Pu. J. Chem. Phys. 2010, 132, 154104 10.1063/1.3382344. 20423165

[ref77] VandeVondele J.; Krack M.; Mohamed F.; Parrinello M.; Chassaing T.; Hutter J. Quickstep: fast and accurate density functional calculations using a mixed Gaussian and plane waves approach. Comput. Phys. Commun. 2005, 167, 103–128. 10.1016/j.cpc.2004.12.014.

[ref78] VandeVondele J.; Hutter J. Gaussian basis sets for accurate calculations on molecular systems in gas and condensed phases. J. Chem. Phys. 2007, 127, 114105 10.1063/1.2770708. 17887826

[ref79] Nosé S. A molecular dynamics method for simulations in the canonical ensemble. Mol. Phys. 1984, 52, 255–268. 10.1080/00268978400101201.

[ref80] Lippert G.; Hutter J.; Parrinello M. The Gaussian and augmented-plane-wave density functional method for ab initio molecular dynamics simulations. Theor. Chem. Acc. 1999, 103, 124–140. 10.1007/s002140050523.

[ref81] Goedecker S.; Teter M.; Hutter J. Separable dual-space Gaussian pseudopotentials. Phys. Rev. B:Condens. Matter Mater. Phys. 1996, 54, 1703–1710. 10.1103/PhysRevB.54.1703. 9986014

[ref82] Hartwigsen C.; Goedecker S.; Hutter J. Relativistic separable dual-space Gaussian pseudopotentials from H to Rn. Phys. Rev. B:Condens. Matter Mater. Phys. 1998, 58, 3641 10.1103/PhysRevB.58.3641. 9986014

