# Supplementary material for: Computational Modeling of the Mobility, Stability, and Al Positioning Ability of Cyclic Cationic Organic Structure-Directing Agents in AEI Zeolite
Source: JACS Au. 2025 Mar 7;5(3):1471–81. doi: 10.1021/jacsau.5c00094 (PMC11938027; doi:10.1021/jacsau.5c00094)
Supplement: Supplementary file 1 — au5c00094_si_001.pdf [file au5c00094_si_001.pdf]

Computational Modeling of the Mobility, Stability and Al Positioning Ability of Cyclic Cationic Organic Structure Directing Agents in AEI Zeolite

Pau Ferri,[1] Pieter Cnudde[2], Manuel Moliner,[1] Veronique van Speybroeck[2,*] and Mercedes Boronat[1,*]

[1] Instituto de Tecnología Química, Universitat Politècnica de València-Consejo Superior de Investigaciones Científicas, Avenida de los Naranjos s/n, 46022 València, Spain

[2] Center for Molecular Modeling, Ghent University, Technologiepark 46, 9052 Zwijnaarde, Belgium

Supporting Information

Table S1. Chemical names of the seven OSDAs considered in this work.

OSDA1	*N,N*-dimethyl-2,6-dimethylpiperidinium
OSDA2	*N,N*-dimethyl-3,5-dimethylpiperidinium
OSDA3	*N,N*-diethyl-2,6-dimethylpiperidinium
OSDA4	*N*-ethyl,*N*-mehyl-2,2,6,6-tetramethylpiperidinium
OSDA5	2,2,7,7-tetramethyl-2-azoniabicyclo[4.1.1]octane
OSDA6	1,3,3,6,6-pentamethyl-6-azoniabicyclo [3.2.1]octane
OSDA7	9,9-dimethyl-9-azabicyclo[3.3.1]nonane

Table S2. Optimized geometries of N-containing cationic OSDAs and the corresponding C-containing neutral OSDA models.

	rN-C (Å)				rC-C (Å)			
OSDA1-*cis*	1.51	1.52	1.50	1.51	1.55	1.56	1.54	1.54
OSDA1-*trans*	1.52	1.53	1.50	1.50	1.55	1.58	1.54	1.54
OSDA2-*cis*	1.52	1.51	1.50	1.50	1.55	1.55	1.54	1.54
OSDA2-*trans*	1.52	1.52	1.50	1.50	1.55	1.54	1.54	1.54
OSDA3-*cis*	1.55	1.56	1.53	1.53	1.57	1.55	1.55	1.55
OSDA3-*trans*	1.55	1.57	1.53	1.53	1.58	1.56	1.55	1.55

Table S3. Absolute interaction energies E_{int} (in kJ/mol) and interaction energies normalized per number of C atoms E_{int}/C (in kJ/mol C) between OSDA1 with ring sizes from 5-C to 10-C and a neutral $Si_{48}O_{96}$ AEI framework obtained from static revPBE-D3 calculations.

	E_{int} (kJ/mol)		E_{int}/C (kJ/mol C)	
	or1	*or2*	*or1*	*or2*
OSDA1-5-C-*cis*	-144	-144	-16	-16
OSDA1-5-C-*trans*	-142	-143	-16	-16
OSDA1-6-C-*cis*	-151	-140	-15	-14
OSDA1-6-C-*trans*	-156	-159	-16	-16
OSDA1-7-C-*cis*	-174	-181	-16	-16
OSDA1-7-C-*trans*	-174	-195	-16	-18
OSDA1-8-C-*cis*	-175	-174	-15	-14
OSDA1-8-C-*trans*	-157	-169	-13	-14
OSDA1-9-C-*cis*	-173	-161	-13	-12
OSDA1-9-C-*trans*	-187	-184	-14	-14
OSDA1-10-C-*cis*	-177	-165	-13	-12
OSDA1-10-C-*trans*	-150	-178	-11	-13

Table S4. Absolute interaction energies E_{int} (in kJ/mol) and interaction energies normalized per number of C atoms E_{int}/C (in kJ/mol C) between OSDA2 or OSDA3 with ring sizes from 5-C to 7-C and a neutral $Si_{48}O_{96}$ AEI framework obtained from static revPBE-D3 calculations.

	E_{int} (kJ/mol)		E_{int}/C (kJ/mol C)	
	or1	*or2*	*or1*	*or2*
OSDA2-5-C-*cis*	-127	-142	-14	-16
OSDA2-5-C-*trans*	-137	-124	-15	-14
OSDA2-6-C-*cis*	-151	-139	-15	-14
OSDA2-6-C-*trans*	-152	-138	-15	-14
OSDA2-7-C-*cis*	-163	-151	-15	-14
OSDA2-7-C-*trans*	-169	-146	-15	-13
OSDA3-5-C-*cis*	-166	-172	-18	-19
OSDA3-5-C-*trans*	-170	-153	-19	-17
OSDA3-6-C-*cis*	-184	-169	-18	-17
OSDA3-6-C-*trans*	-171	-197	-17	-20
OSDA3-7-C-*cis*	-182	-187	-17	-17
OSDA3-7-C-*trans*	-192	-189	-17	-17

Table S5. Optimized Al-N distance (in Å) for all structures obtained from static revPBE-D3 calculations for each cationic OSDA inside the AEI framework with Al siting at T1, T2 and T3.

	T1			T2			T3		
	or1	*or2*	*or3*	*or1*	*or2*	*or3*	*or1*	*or2*	*or3*
OSDA1	5,2	4,9	4,9	6,5	5,8	5,8	5,1	5,4	4,7
OSDA2	5,3	7,2	4,8	7,1	6,8	5,6	5,2	6,7	5,0
OSDA3	5,4	5,8	5,8	6,9	6,6	6,6	5,2	5,7	5,8
OSDA4	5,0	5,0	4,8	6,1	5,7	5,6	5,2	7,4	5,4
OSDA5	5,4	4,9	4,8	7,0	5,8	5,5	5,3	4,8	4,9
OSDA6	5,2	4,8	4,7	7,1	5,4	5,1	5,0	5,3	4,7
OSDA7	4,6	5,0	4,8	5,6	5,7	5,6	5,5	5,6	5,4

Table S6. Relative stability (in kJ/mol) of all structures obtained from static revPBE-D3 calculations for each cationic OSDA inside the AEI framework with Al siting at T1, T2 and T3.

	T1			T2			T3		
	or1	*or2*	*or3*	*or1*	*or2*	*or3*	*or1*	*or2*	*or3*
OSDA1	5	**0**	16	19	13	29	7	8	18
OSDA2	5	38	**0**	22	64	13	15	65	10
OSDA3	**0**	4	20	19	33	27	7	24	28
OSDA4	**0**	37	16	12	51	16	7	57	16
OSDA5	25	14	**0**	36	25	22	23	19	9
OSDA6	21	6	**0**	36	14	34	29	16	5
OSDA7	5	2	3	19	9	15	6	**0**	16

Table S7. Interaction energies E_{int} (in kJ/mol) between cationic OSDAs and the negatively charged $AlSi_{47}O_{96}$ AEI framework obtained from static revPBE-D3 calculations. The lowest E_{int} value for each OSDA is highlighted (bold letter)

	T1			T2			T3		
	or1	*or2*	*or3*	*or1*	*or2*	*or3*	*or1*	*or2*	*or3*
OSDA1-5-C-*cis*	**-653**	-649	-648	-645	-621	-642	-631	-618	-646
OSDA1-5-C-*trans*	**-661**	-649	-647	-650	-645	-640	-649	-642	-642
OSDA1-6-C-*cis*	-635	**-640**	-624	-627	-632	-617	-636	-636	-626
OSDA1-6-C-*trans*	**-642**	**-642**	-631	-629	-637	-623	-640	-636	-627
OSDA1-7-C-*cis*	-677	**-693**	-683	-666	-686	-668	-673	-683	-680
OSDA1-7-C-*trans*	-651	-675	-665	-641	-664	-655	-647	**-679**	-662
OSDA2-5-C-*cis*	**-661**	-654	-659	-649	-649	-659	-655	-647	-659
OSDA2-5-C-*trans*	**-651**	-640	-649	-637	-638	-647	-645	-634	-646
OSDA2-6-C-*cis*	-663	-629	**-668**	-651	-609	-662	-656	-606	-661
OSDA2-6-C-*trans*	**-666**	-647	-664	-650	-644	-659	-660	-639	-659
OSDA2-7-C-*cis*	**-671**	-647	-667	-661	-645	-645	-665	-638	-663
OSDA2-7-C-*trans*	-678	-673	**-679**	-665	-667	-677	-672	-664	-652

Table S8. Optimized Al-N distance (in Å) for all structures in Table S6, obtained from static revPBE-D3 calculations for each cationic OSDA inside the AEI framework with Al siting at T1, T2 and T3.

	T1			T2			T3		
	or1	*or2*	*or3*	*or1*	*or2*	*or3*	*or1*	*or2*	*or3*
OSDA1-5-C-*cis*	5,3	5,7	5,0	6,6	5,9	5,9	5,1	5,4	5,1
OSDA1-5-C-*trans*	5,4	5,2	5,1	6,5	5,8	5,9	5	5.8	5
OSDA1-6-C-*cis*	**5,2**	4,9	4,9	6,5	5,8	5,8	5,1	5,4	4,7
OSDA1-6-C-*trans*	5,2	5,0	5,0	6,4	5,8	5,8	4,9	5,7	4,7
OSDA1-7-C-*cis*	5,2	4,7	4,9	6,6	5,7	6,3	5,2	5,9	4,8
OSDA1-7-C-*trans*	5,2	4.8	5,1	6,5	5,8	6,1	4,8	5,7	4,8
OSDA2-5-C-*cis*	5,1	5,8	5,3	6,8	6,1	6	4,9	5,4	4,8
OSDA2-5-C-*trans*	5,3	5,8	5,2	6,7	6,2	5,7	5	5,6	5
OSDA2-6-C-*cis*	5,3	7,2	**4,8**	7,1	6,8	5,6	5,2	6,7	5,0
OSDA2-6-C-*trans*	5	7,4	5	6,4	5,9	6,8	5,1	5,4	4,8
OSDA2-7-C-*cis*	5,2	7,3	5,2	6,1	5,8	6,6	4,8	5,4	4,7
OSDA2-7-C-*trans*	5	7,1	5,1	6,3	5,9	6,4	4,8	5,6	4,8

Table S9. Calculated N-Al distances (Å) from static revPBE-D3 optimizations and averaged N-Al distances (Å) from 75 ps AIMD runs for different OSDAs in different initial orientations.

		T1		T2		T3	
		Static	AIMD	Static	AIMD	Static	AIMD
OSDA1	*or1*	**5,2**	5.4	6,5	6.8	5,1	5.2
	or2	4,9	5.3	5,8	6.1	5,4	5.7
OSDA2	*or1*	5,3	5.3	7,1	7.2	5,2	5.0
	or2	5,1	5.1	6,8	7.1	6,7	6.6
	or3	**4,8**	5.1	5,6	5.8	5,0	5.1
OSDA5	*or3*	4,8	5.3	5,5	5.8	4,9	5.2
OSDA6	*or3*	4,7	5.0	5,1	5.6	4,7	4.5
OSDA7	*or1*	4,6	5.0	5,6	6.5	5,5	5.1
	or2	5,0	5.2	5,7	6	5,6	5.0
	or3	4,8	5.2	5,6	5.8	5,4	5.3
	or4	5,5	5.1	6,2	6.3	5,3	5.6
	or5	4,9	5.1	6,5	6.6	6,1	5.3
	or6	5,2	5.3	6,5	6.5	6,0	5.2
	or7	6,2	5.2	6,6	6.2	5,4	5.2

Table S10. Interaction energies E_{int} (in kJ/mol) between cationic OSDA7 in seven different orientations and the negatively charged $AlSi_{47}O_{96}$ AEI framework obtained from static revPBE-D3 calculations.

	T1	T2	T3
or1	-663	-655	-667
or2	-667	-665	**-672**
or3	-666	-659	-657
or4	-658	-653	-652
or5	-667	-658	-664
or6	-665	-659	-661
or7	-648	-646	-640

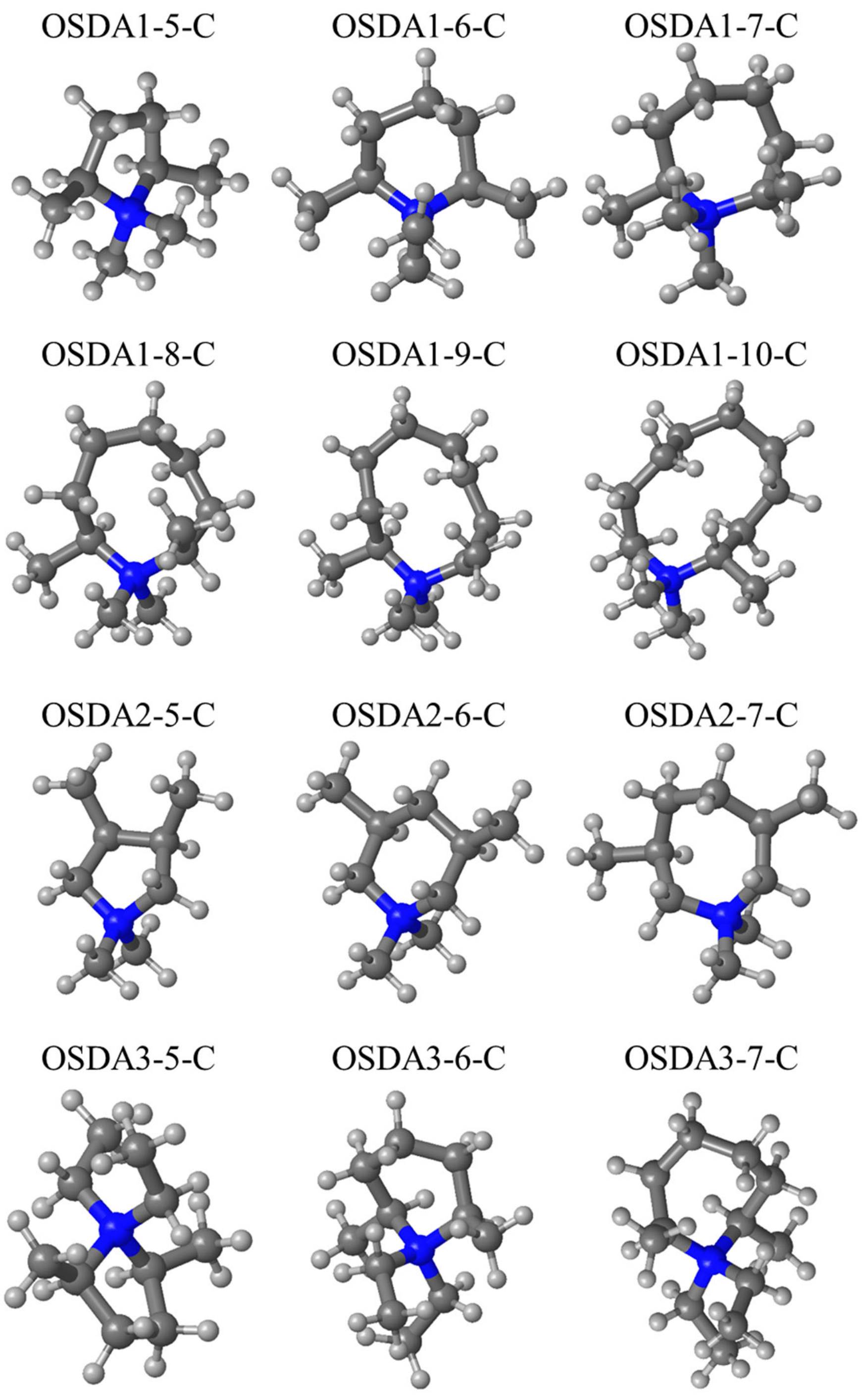

Figure S1. Set of piperidinium-based OSDAs with increasing ring-size studied in this work. N, C and H atoms depicted as blue, grey and white balls, respectively.

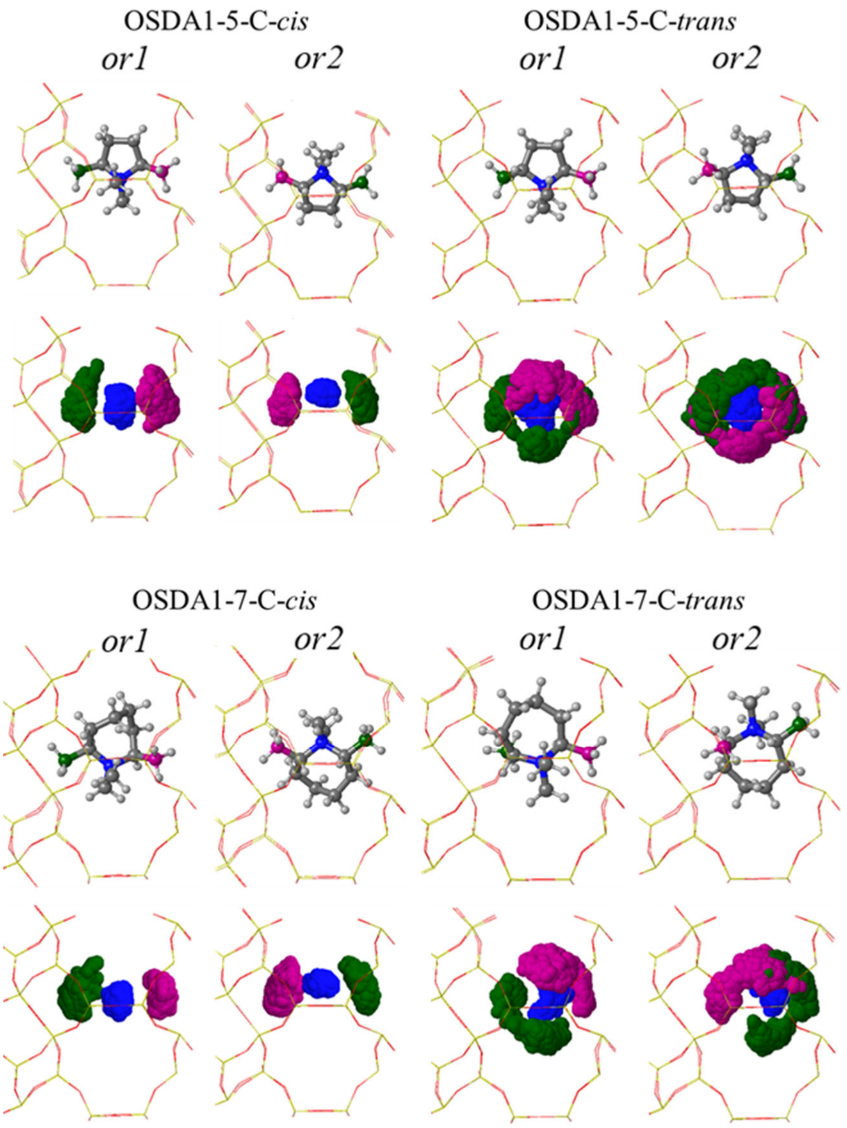

Figure S2. Initial geometries and scatterplots for the "N" (blue) and methyl ring-substituents (green and purple) for OSDA1-5-C and OSDA1-7-C obtained from 75 ps AIMD simulations at 408 K. Framework Si and O atoms depicted as yellow and red sticks. "N", C and H atoms depicted as blue, grey and white balls.

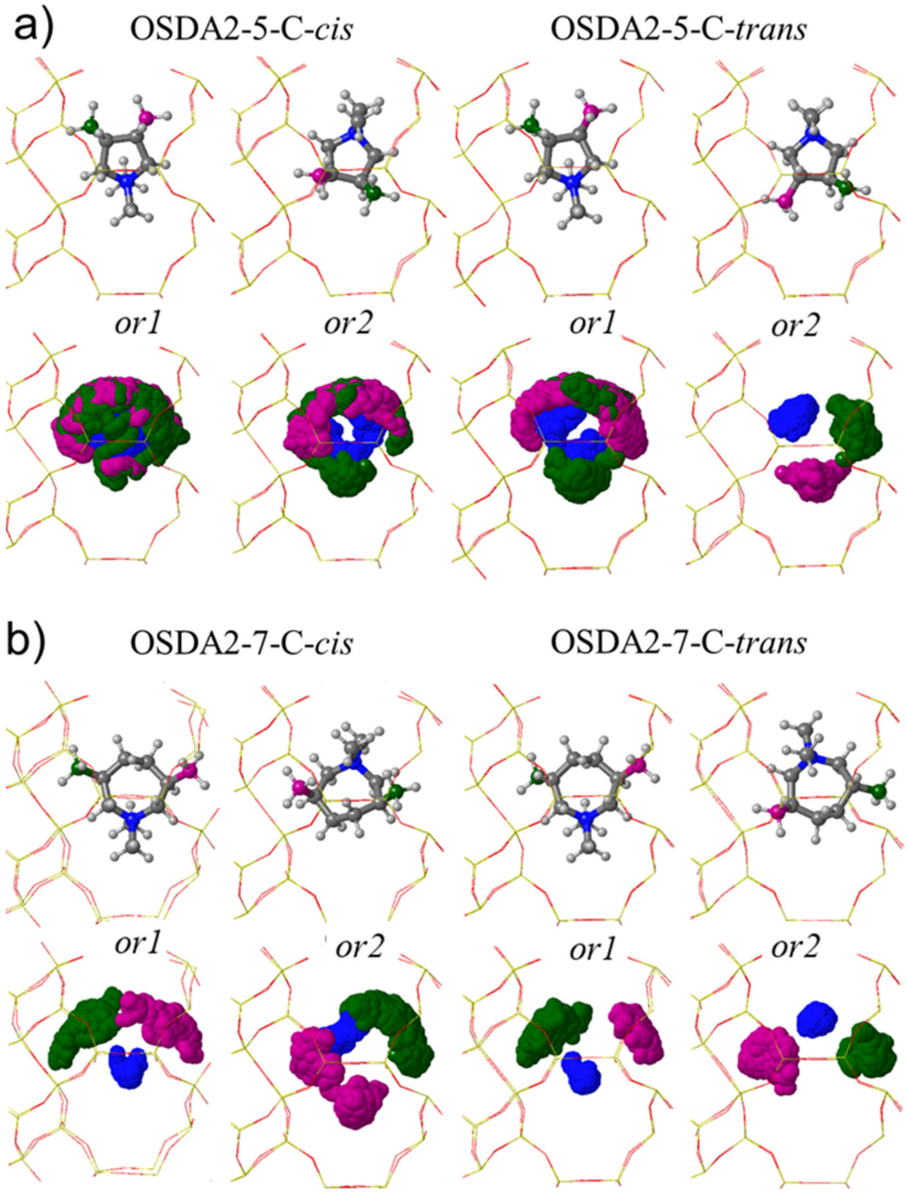

Figure S3. Initial geometries and scatterplots for the “N” (blue) and methyl ring-substituents (green and purple) for OSDA2-5-C (a) and OSDA2-7-C (b) obtained from 75 ps AIMD simulations at 408 K. Framework Si and O atoms depicted as yellow and red sticks. “N”, C and H atoms depicted as blue, grey and white balls.

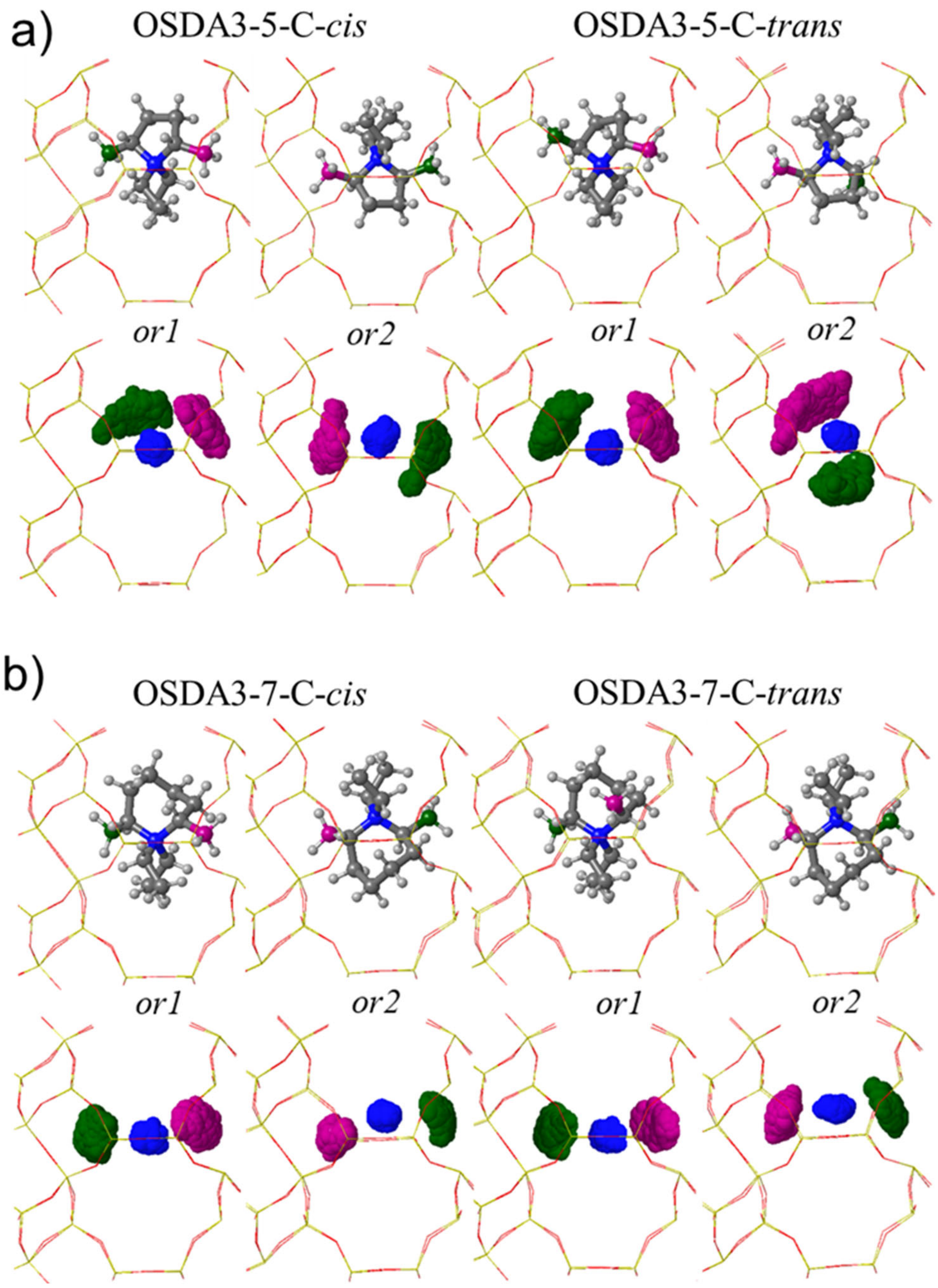

Figure S4. Initial geometries and scatterplots for the "N" (blue) and methyl ring-substituents (green and purple) for OSDA3-5-C (a) and OSDA3-7-C (b) obtained from 75 ps AIMD simulations at 408 K. Framework Si and O atoms depicted as yellow and red sticks. "N", C and H atoms depicted as blue, grey and white balls.

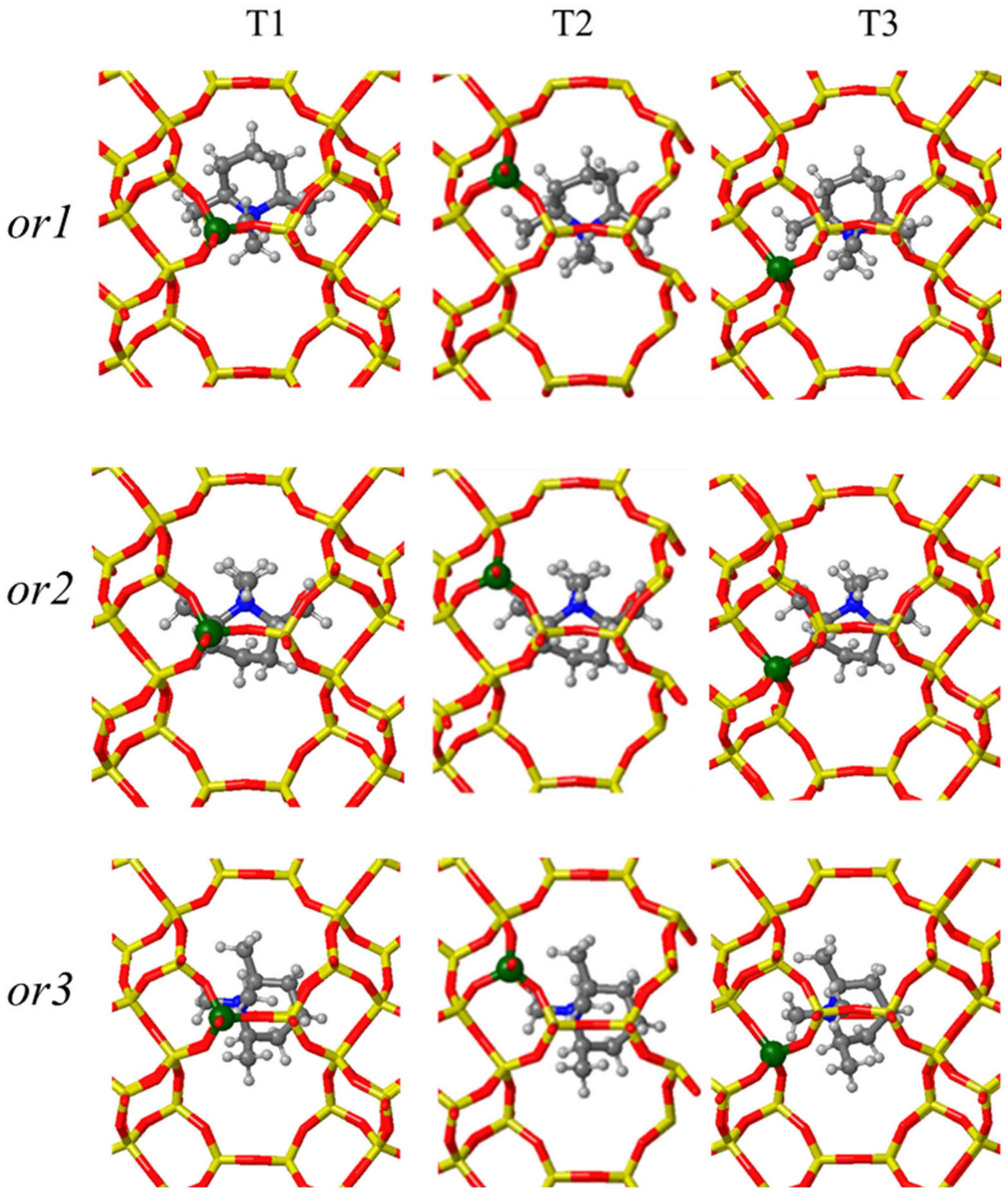

Figure S5. Optimized geometries of OSDA1 stabilized within an AEI cavity containing one Al atom in each of the three T1, T2 and T3 positions, obtained from static DFT-D3 calculations. Framework Si and O atoms depicted as yellow and red sticks. Al, N, C and H atoms depicted as green, blue, grey and white balls.

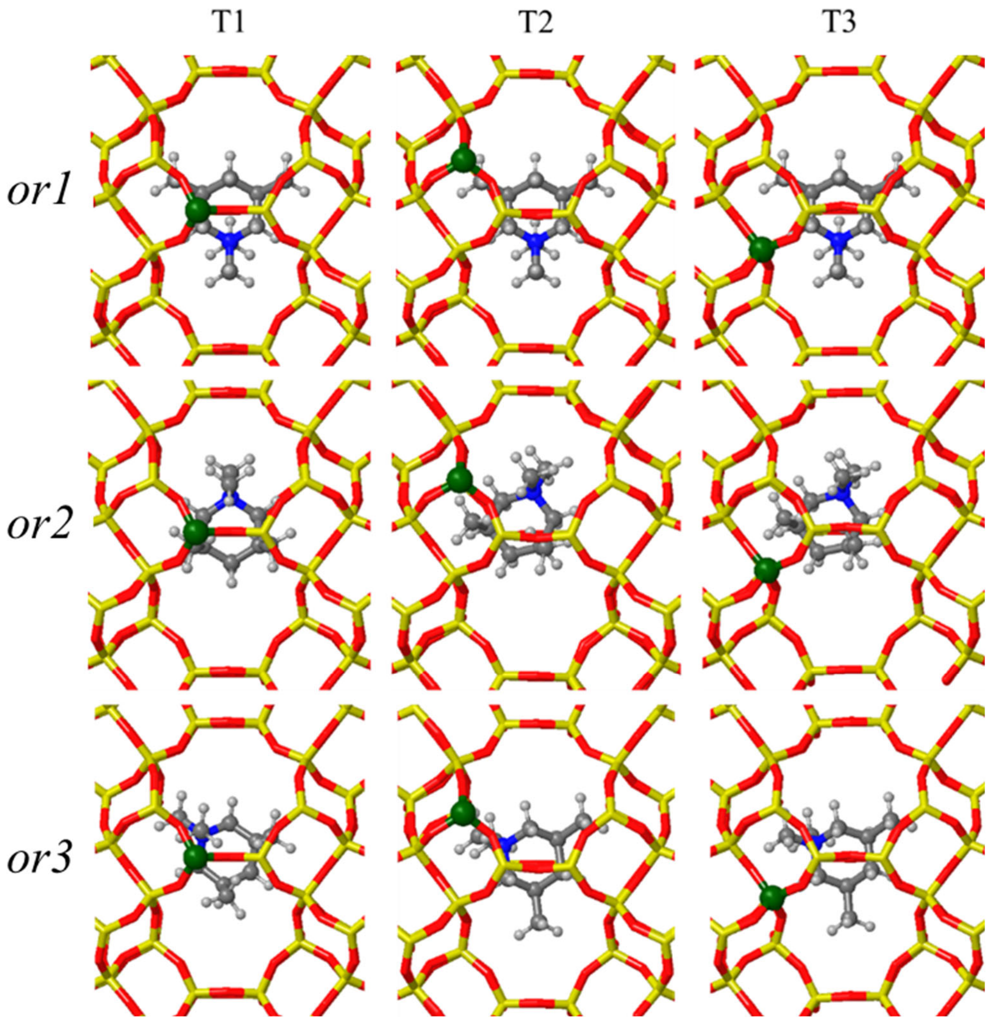

Figure S6. Optimized geometries of OSDA2 stabilized within an AEI cavity containing one Al atom in each of the three T1, T2 and T3 positions, obtained from static DFT-D3 calculations. Framework Si and O atoms depicted as yellow and red sticks. Al, N, C and H atoms depicted as green, blue, grey and white balls.

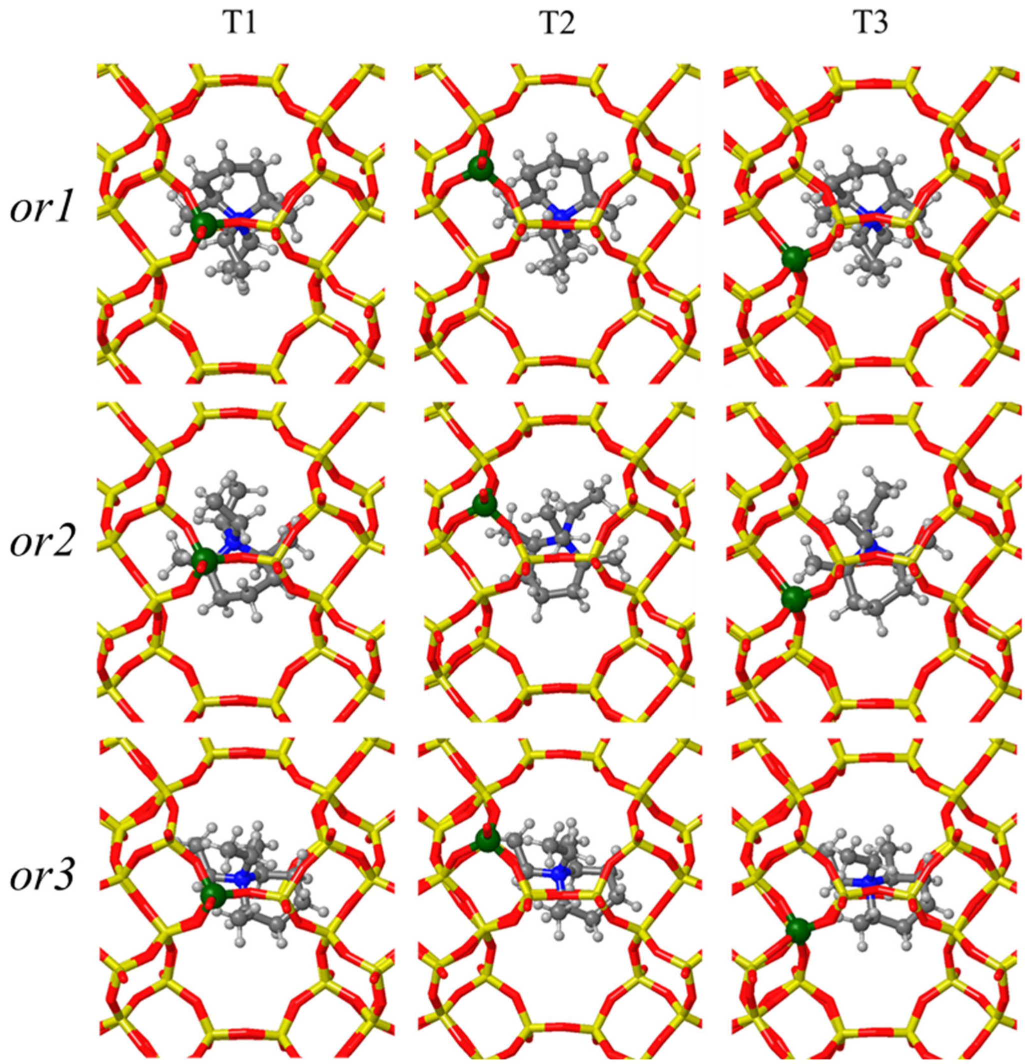

Figure S7. Optimized geometries of OSDA3 stabilized within an AEI cavity containing one Al atom in each of the three T1, T2 and T3 positions, obtained from static revPBE-D3 calculations. Framework Si and O atoms depicted as yellow and red sticks. Al, N, C and H atoms depicted as green, blue, grey and white balls.

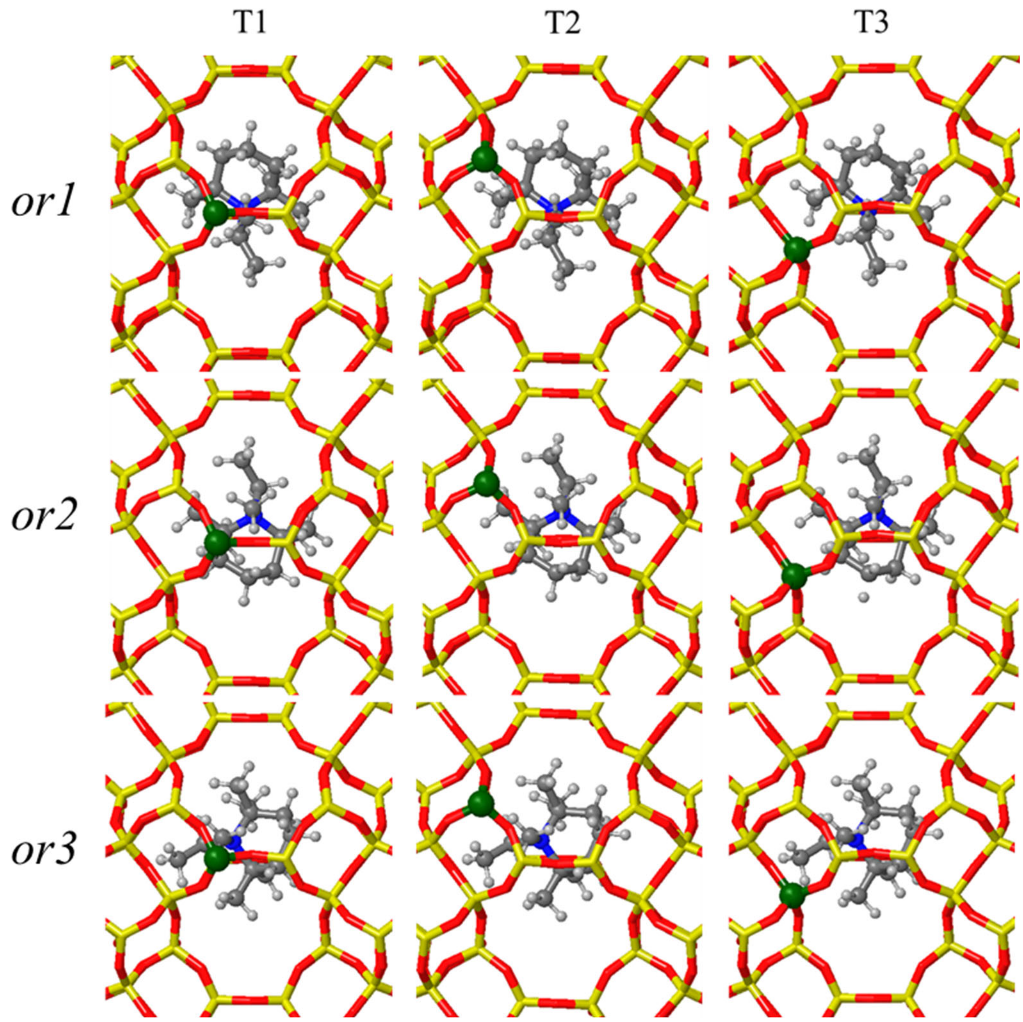

Figure S8. Optimized geometries of OSDA4 stabilized within an AEI cavity containing one Al atom in each of the three T1, T2 and T3 positions, obtained from static revPBE-D3 calculations. Framework Si and O atoms depicted as yellow and red sticks. Al, N, C and H atoms depicted as green, blue, grey and white balls.

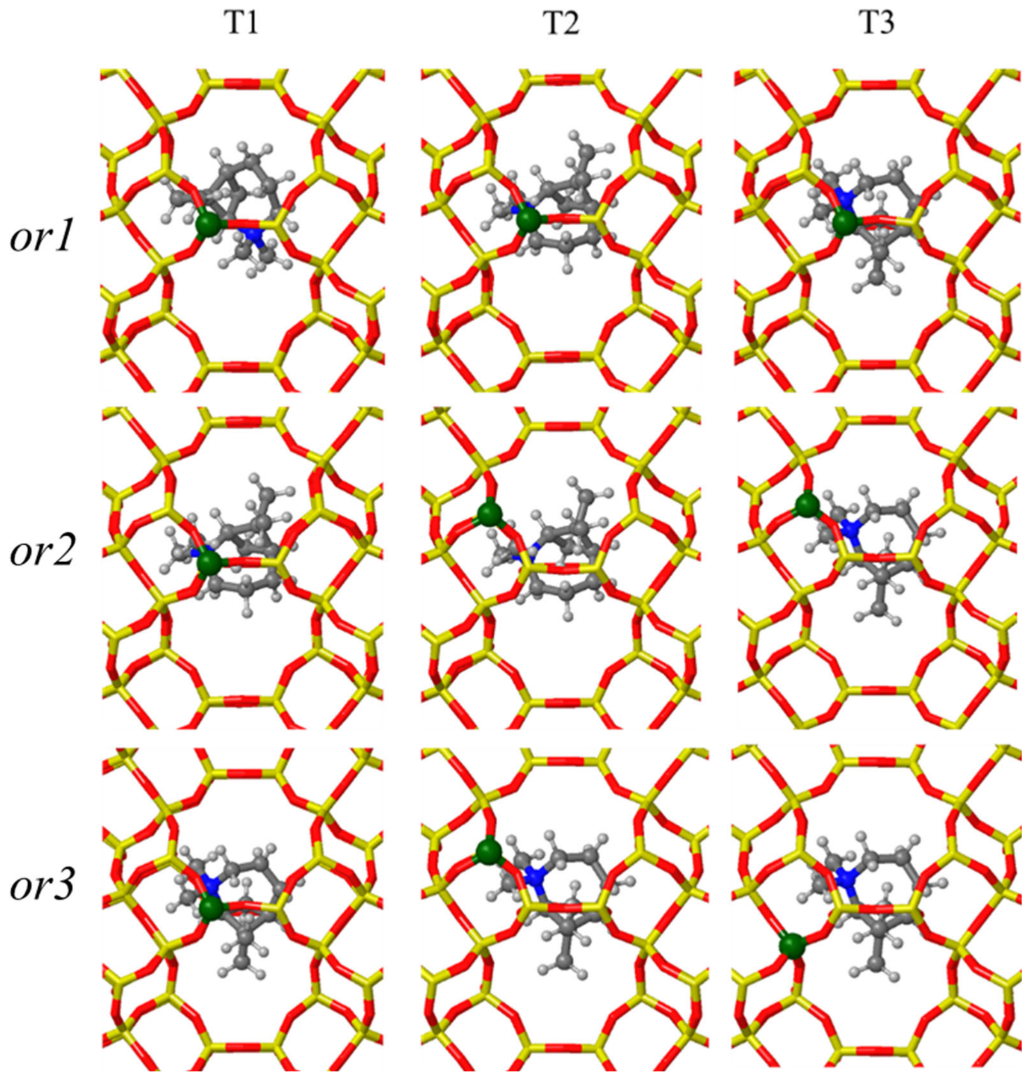

Figure S9. Optimized geometries of OSDA5 stabilized within an AEI cavity containing one Al atom in each of the three T1, T2 and T3 positions, obtained from static revPBE-D3 calculations. Framework Si and O atoms depicted as yellow and red sticks. Al, N, C and H atoms depicted as green, blue, grey and white balls.

.

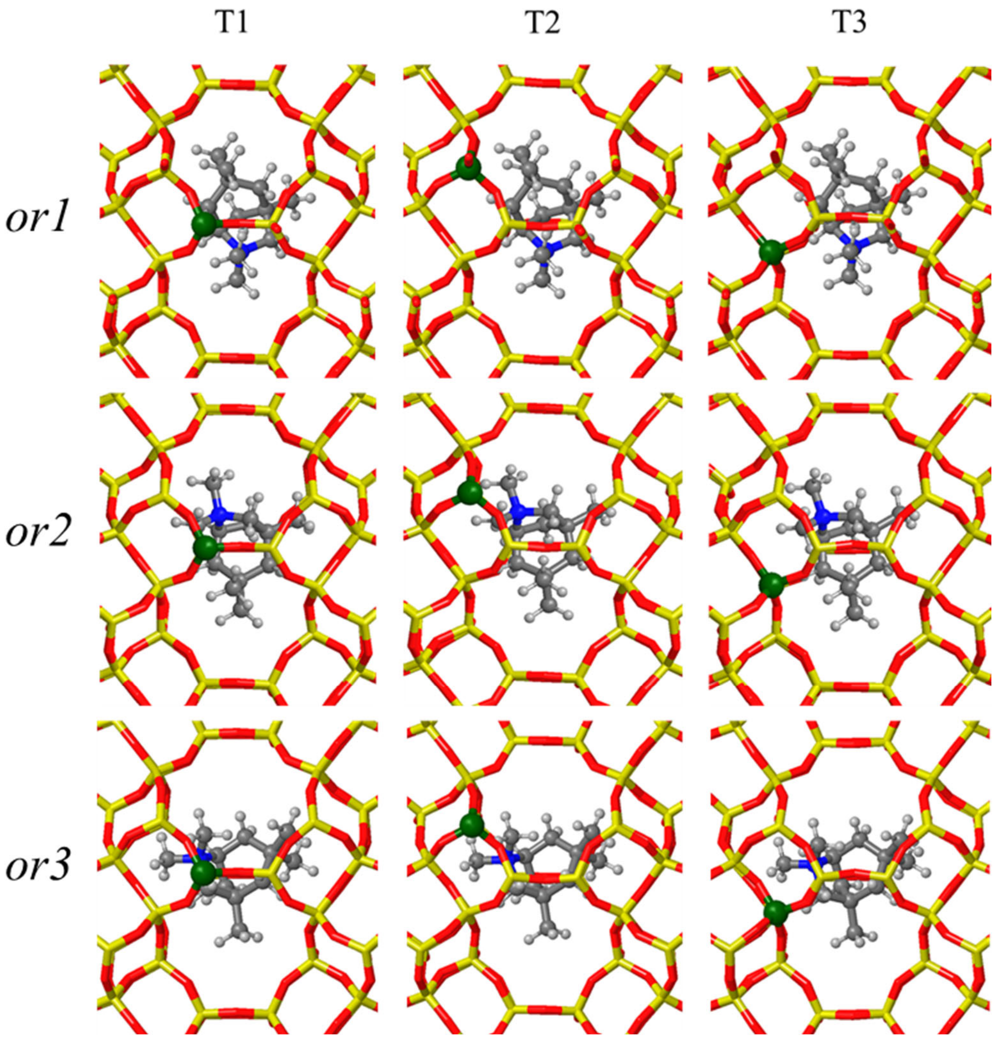

Figure S10. Optimized geometries of OSDA6 stabilized within an AEI cavity containing one Al atom in each of the three T1, T2 and T3 positions, obtained from static revPBE-D3 calculations. Framework Si and O atoms depicted as yellow and red sticks. Al, N, C and H atoms depicted as green, blue, grey and white balls.

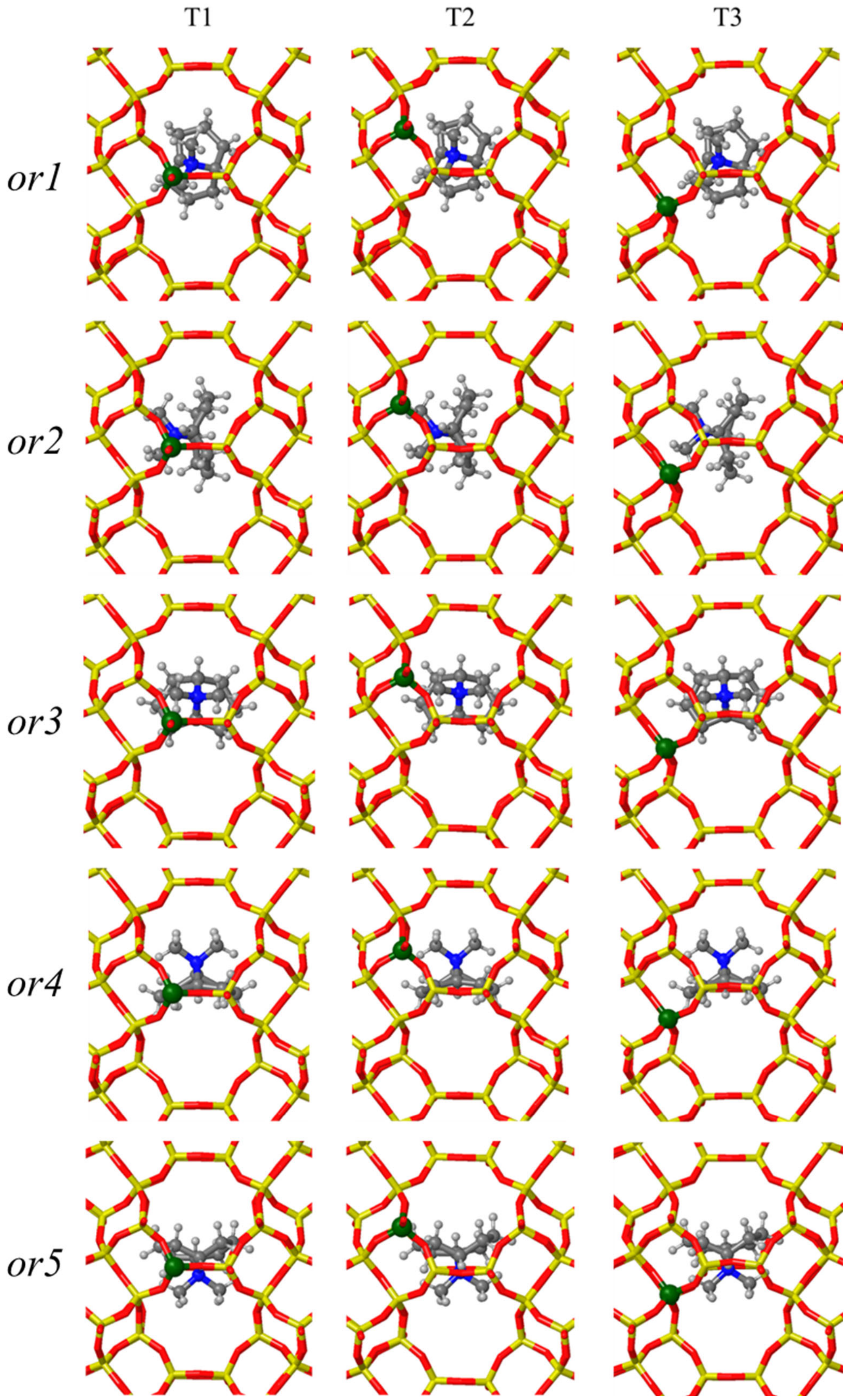
T1
T2
T3
or1
or2
or3
or4
or5

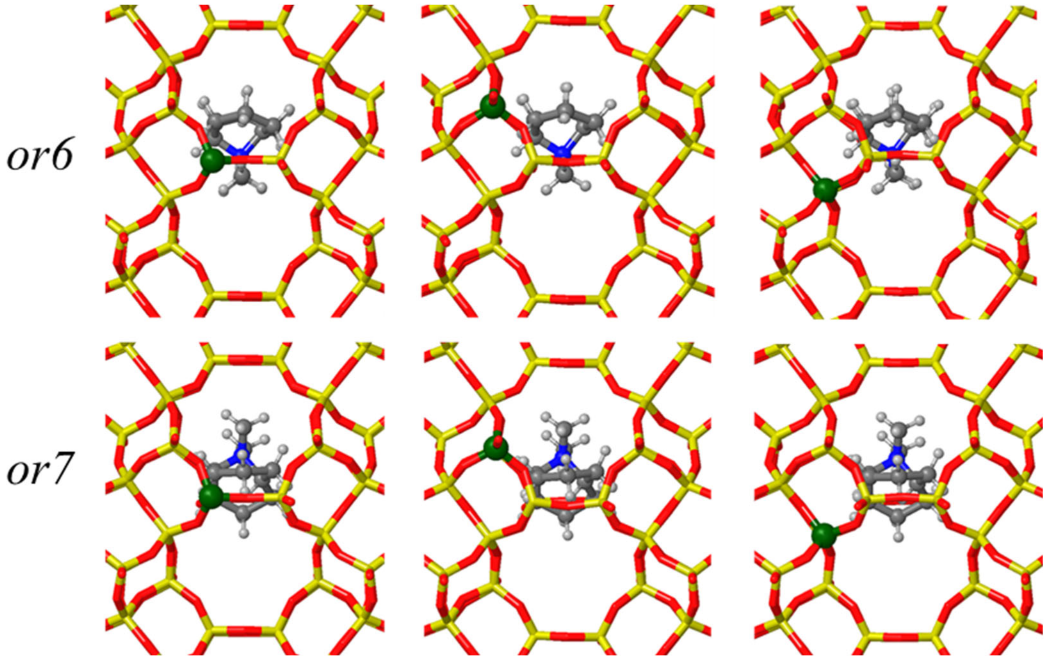

Figure S11. Optimized geometries of OSDA7 stabilized within an AEI cavity containing one Al atom in each of the three T1, T2 and T3 positions, obtained from static revPBE-D3 calculations. Framework Si and O atoms depicted as yellow and red sticks. Al, N, C and H atoms depicted as green, blue, grey and white balls.

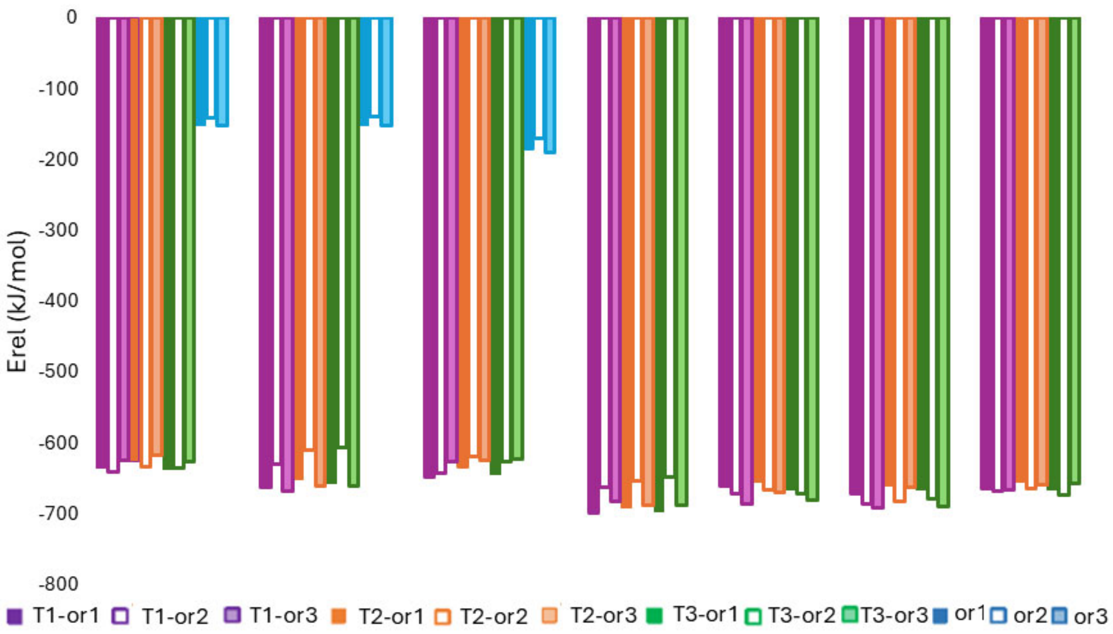

Figure S12. Interaction revPBE-D3 energies E_{int} (in kJ/mol) between cationic OSDAs in three different orientations (*or1*, *or2*, *or3*) and the negatively charged $AlSi_{47}O_{96}$ AEI framework with Al in three different crystallographic positions (T1, T2, T3) obtained from static PBE-D3 calculations (purple, orange and green bars). The interaction energies E_{int} (in kJ/mol) between neutral OSDAs and the neutral $Si_{48}O_{96}$ AEI framework are plotted as blue bars for comparison.

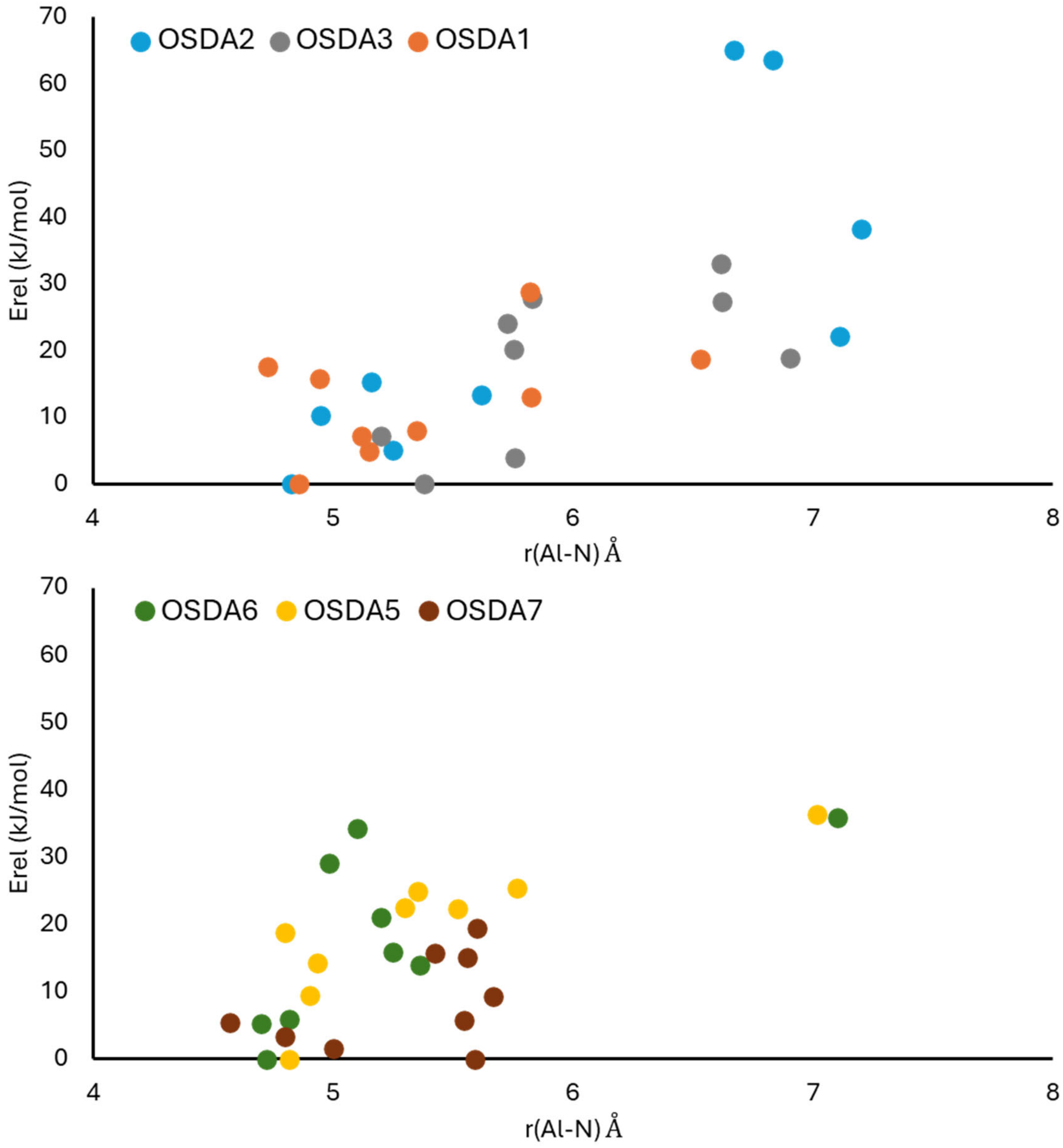

Figure S13. Plots of the relative revPBE-D3 interaction energies (in kJ/mol) summarized in Table S6 versus the optimized Al-N distances (in Å) given in Table S5.

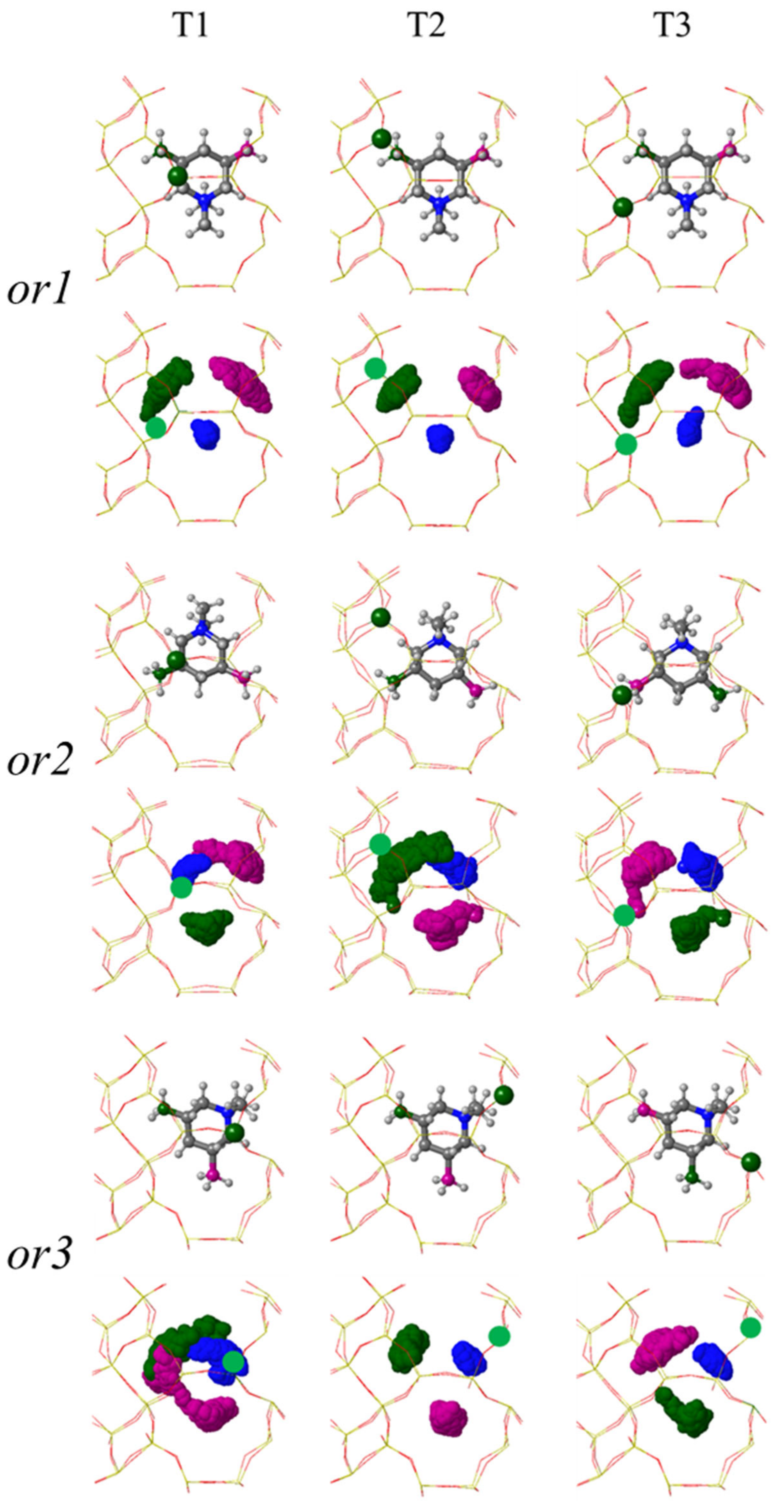

Figure S14. Initial geometries and scatterplots for the N atom (blue) and methyl ring-substituents (green and purple) for OSDA2 in an AEI cavity containing one Al atom in each of the three T1, T2 and T3 positions, obtained from 75 ps AIMD simulations at 408 K. Framework Si and O atoms depicted as yellow and red sticks. Al, N, C and H atoms depicted as green, blue, grey and white balls. For clarity, Al is depicted as light green circles in the scatterplots.

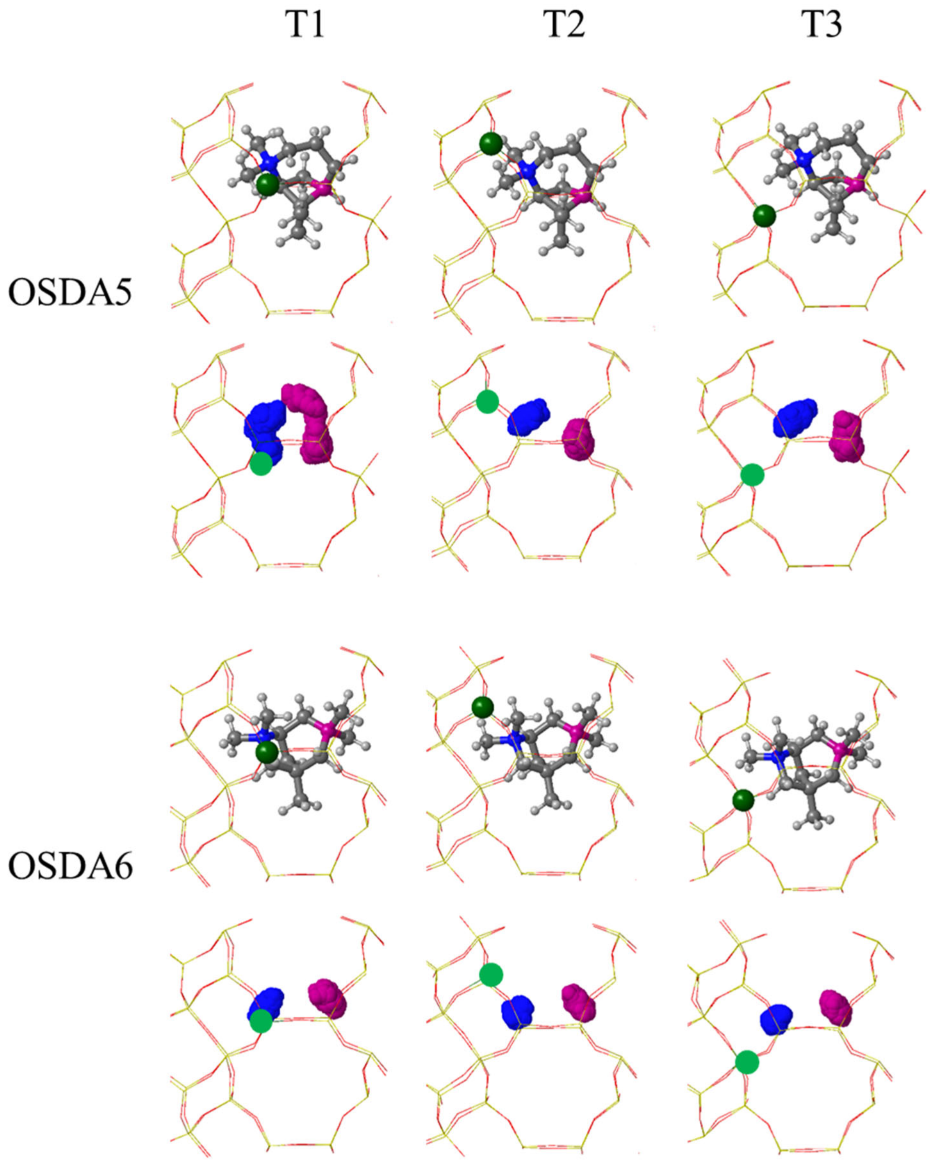

Figure S15. Initial geometries and scatterplots for the N atom (blue) and one methyl ring-substituent (purple) for OSDA5 and OSDA6 in an AEI cavity containing one Al atom in each of the three T1, T2 and T3 positions, obtained from 75 ps AIMD simulations at 408 K. Framework Si and O atoms depicted as yellow and red sticks. Al, N, C and H atoms depicted as green, blue, grey and white balls. For clarity, Al is depicted as light green circles in the scatterplots.

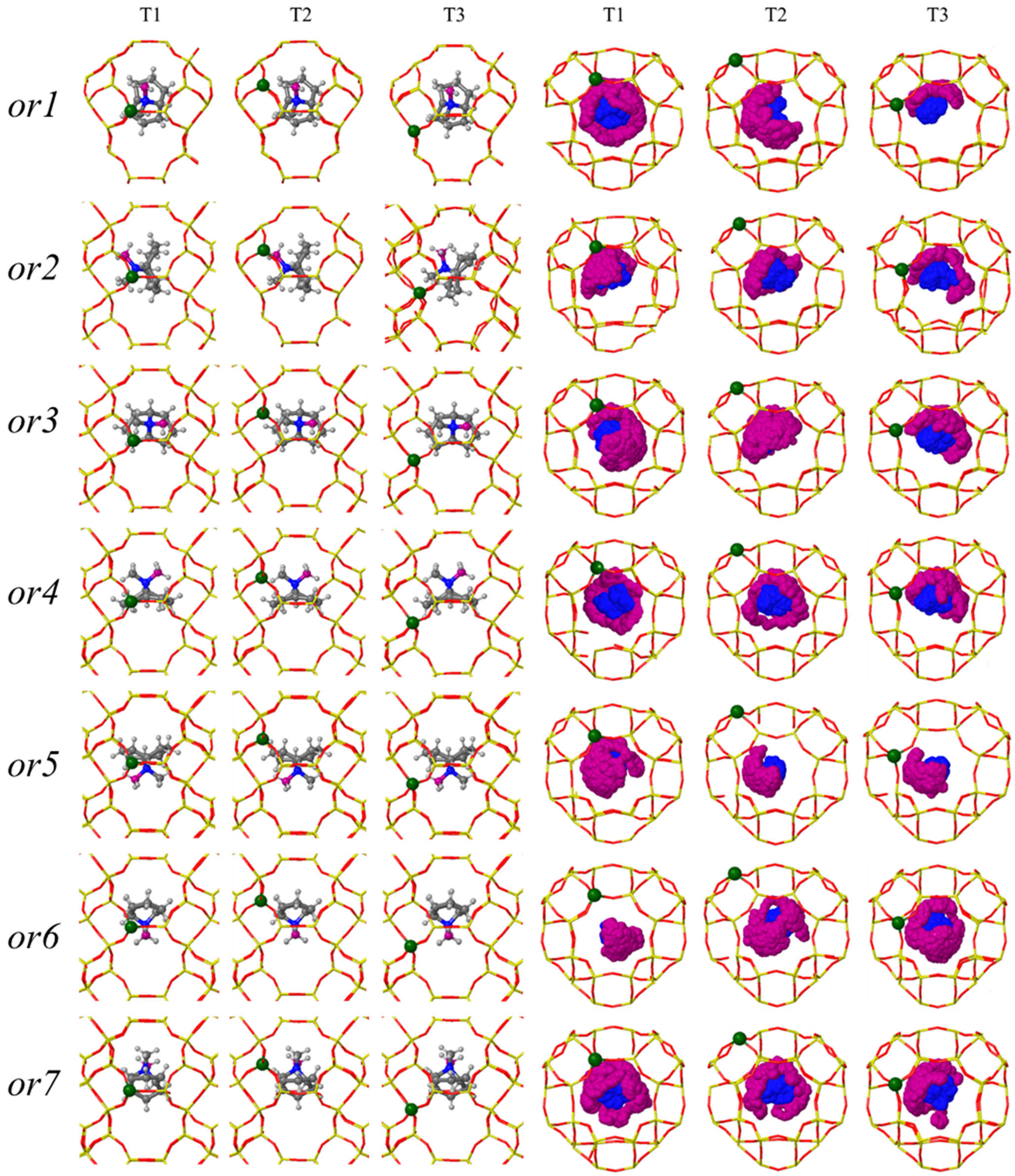

Figure S16. Initial geometries and scatterplots for the N atom (blue) and one methyl ring-substituent (purple) for OSDA7 in an AEI cavity containing one Al atom in each of the three T1, T2 and T3 positions, obtained from 75 ps AIMD simulations at 408 K. Framework Si and O atoms depicted as yellow and red sticks. Al, N, C and H atoms depicted as green, blue, grey and white balls.

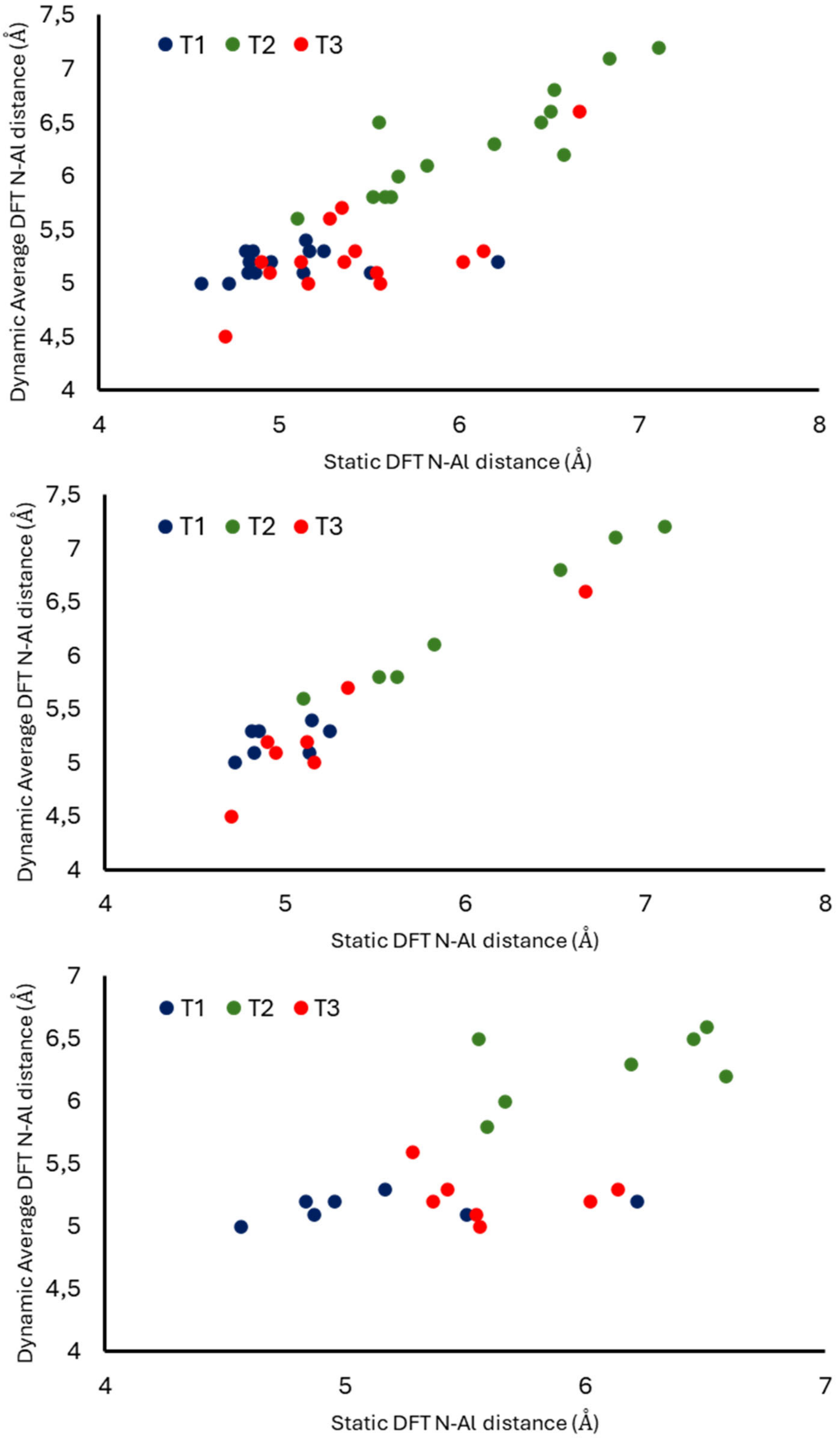

Figure S17. Correlation between the dynamic averaged N-Al distances obtained from AIMD simulations and the optimized N-Al distances obtained from static revPBE-D3 calculations for (a) all structures in this work, (b) all structures except OSDA7 and (c) OSDA7.
